# Melatonin for premenstrual syndrome: A potential remedy but not ready

**DOI:** 10.3389/fendo.2022.1084249

**Published:** 2023-01-09

**Authors:** Wei Yin, Jie Zhang, Yao Guo, Zhibing Wu, Can Diao, Jinhao Sun

**Affiliations:** ^1^ Shandong Key Laboratory of Mental Disorders, Department of Anatomy and Neurobiology, Shandong University, Jinan, Shandong, China; ^2^ Department of Neurosurgery, Laizhou City People’s Hospital, Laizhou, Shandong, China; ^3^ Department of Psychiatry, Shandong Provincial Mental Health Center, Jinan, Shandong, China; ^4^ Department of Anatomy, Changzhi Medical College, Changzhi, Shanxi, China; ^5^ School of Basic Medical Sciences, Cheeloo College of Medicine, Shandong University, Jinan, Shandong, China

**Keywords:** melatonin, premenstrual syndrome, circadian rhythms, ovarian steroid, cognition, gamma-aminobutyric acid

## Abstract

Premenstrual syndrome (PMS), a recurrent and moderate disorder that occurs during the luteal phase of the menstrual cycle and quickly resolves after menstruation, is characterized by somatic and emotional discomfort that can be severe enough to impair daily activities. Current therapeutic drugs for PMS such as selective serotonin reuptake inhibitors are not very satisfying. As a critical pineal hormone, melatonin has increasingly been suggested to modulate PMS symptoms. In this review, we update the latest progress on PMS-induced sleep disturbance, mood changes, and cognitive impairment and provide possible pathways by which melatonin attenuates these symptoms. Moreover, we focus on the role of melatonin in PMS molecular mechanisms. Herein, we show that melatonin can regulate ovarian estrogen and progesterone, of which cyclic fluctuations contribute to PMS pathogenesis. Melatonin also modulates gamma-aminobutyric acid and the brain-derived neurotrophic factor system in PMS. Interpreting the role of melatonin in PMS is not only informative to clarify PMS etiology but also instructive to melatonin and its receptor agonist application to promote female health. As a safe interaction, melatonin treatment can be effective in alleviating symptoms of PMS. However, symptoms such as sleep disturbance, depressive mood, cognitive impairment are not specific and can be easily misdiagnosed. Connections between melatonin receptor, ovarian steroid dysfunction, and PMS are not consistent among past studies. Before final conclusions are drawn, more well-organized and rigorous studies are recommended.

## Introduction

Premenstrual syndrome (PMS) is a kind of neuroendocrine disorder that threatens women’s physical and mental health. It is estimated that more than half of women complain of somatic or emotional discomfort during the luteal phase of the menstrual cycle. In general, these symptoms are mild, and some physical disturbances, such as abdominal swelling and breast tenderness, and mood changes, such as irritability and anxiety, are commonly reported (1–3). However, approximately 5.3% of women experience distress that is too severe, such as insomnia, depression and cognitive impairment, to accomplish daily activities (**Table 1**). This severe form of PMS is defined as premenstrual dysphoric disorder (PMDD) (5). Although the etiology of PMS is not fully illustrated, ovarian hormone fluctuations are clearly associated with PMS, as the symptoms are only observed in the luteal phase and reduced after menstruation. Consequently, in recent years, dysfunctional reproductive hormone levels and their effects on the brain neurotransmitter system have been regarded as key factors in PMS pathogenesis (5, 14, 15). In line with this notion, ovarian cyclicity disruption and brain neurotransmitter regulation have been developed as treatment methods for PMS. Serotonin reuptake inhibitors (SSRIs) are recommended as the first-line therapy for PMDD management (5). However, first-line medications fail to completely relieve symptoms in almost 75% of PMDD patients. As a result, other therapeutic drugs with proof of molecular mechanisms and clinical trials are needed (2). To our knowledge, the therapeutic effect of two or more medicine combinations is always better than that of one medicine alone. For example, oral contraceptives, such as ethinylestradiol drospirenone, can further improve the management of symptoms other than depressive symptoms by suppressing the hypothalamic-pituitary-ovarian axis in PMS and PMDD (16). Ulipristal acetate, a selective progesterone receptor modulator, may be the key to alleviating psychological symptoms such as depression (17).

**Table 1:** Clinical manifestations of premenstrual syndrome.

Clinical manifestation	Disorders with similar clinical presentatation	Reference
Sleep disturbance (poor sleep, insomnia, sleepiness)	Infertility, polycystic ovary syndrome, unexplained sleep dysfunction	(4)
Depression, anxiety	Primary psychiateric issues; unexplained emotional changes	(5)
Cognitve impairment	Alzheimer's disease; vascular dementia; functional cognitive disorders	(6, 7)
Ovarian hormone imbalance	Infertility; endometriosis; perimenopausal syndrome	(8, 9)
GABA-GABAAR activity ↓ (cingulate cortex, medial prefrontal cortex and left basal ganglia)	Psychiatric disorders; decreased sociability; panic disorder	(10, 11)
Alterations in BDNF levels	Depression; schizophrenia; neurodegenerative diseases; brain cancer	(12, 13)

↓ means reduced.

Melatonin is produced from the pineal gland and serves as an internal synchronizer under the tight control of the central circadian timing system (internal clock) located in the suprachiasmatic nucleus (SCN) within the anterior hypothalamus (18). Three decades ago, Parry and his colleagues found a phase-advanced offset of melatonin secretion in PMS (19). A subsequent study demonstrates an altered phase-shift response of melatonin to light in PMDD patients, whereas the suppressive effects of light on melatonin between PMDD and healthy women are similar, indicating the contribution of circadian clock dysfunction in PMDD (20, 21). Melatonin also reciprocally modulates the circadian clock *via* the melatonin receptors MT1 and MT2 (22, 23). In addition, several studies also report that nocturnal melatonin changes are accompanied by sleep, emotion and ovarian hormone alterations and that an exogenous supplement of melatonin can partially correct them (24, 25). More importantly, progressive advances have shown melatonin’s improvement of reproductive functions and neuroprotection (26, 27). These studies suggest that blunted circadian melatonin might contribute to PMS and imply a potential therapeutic effect.

**Figure 1:**
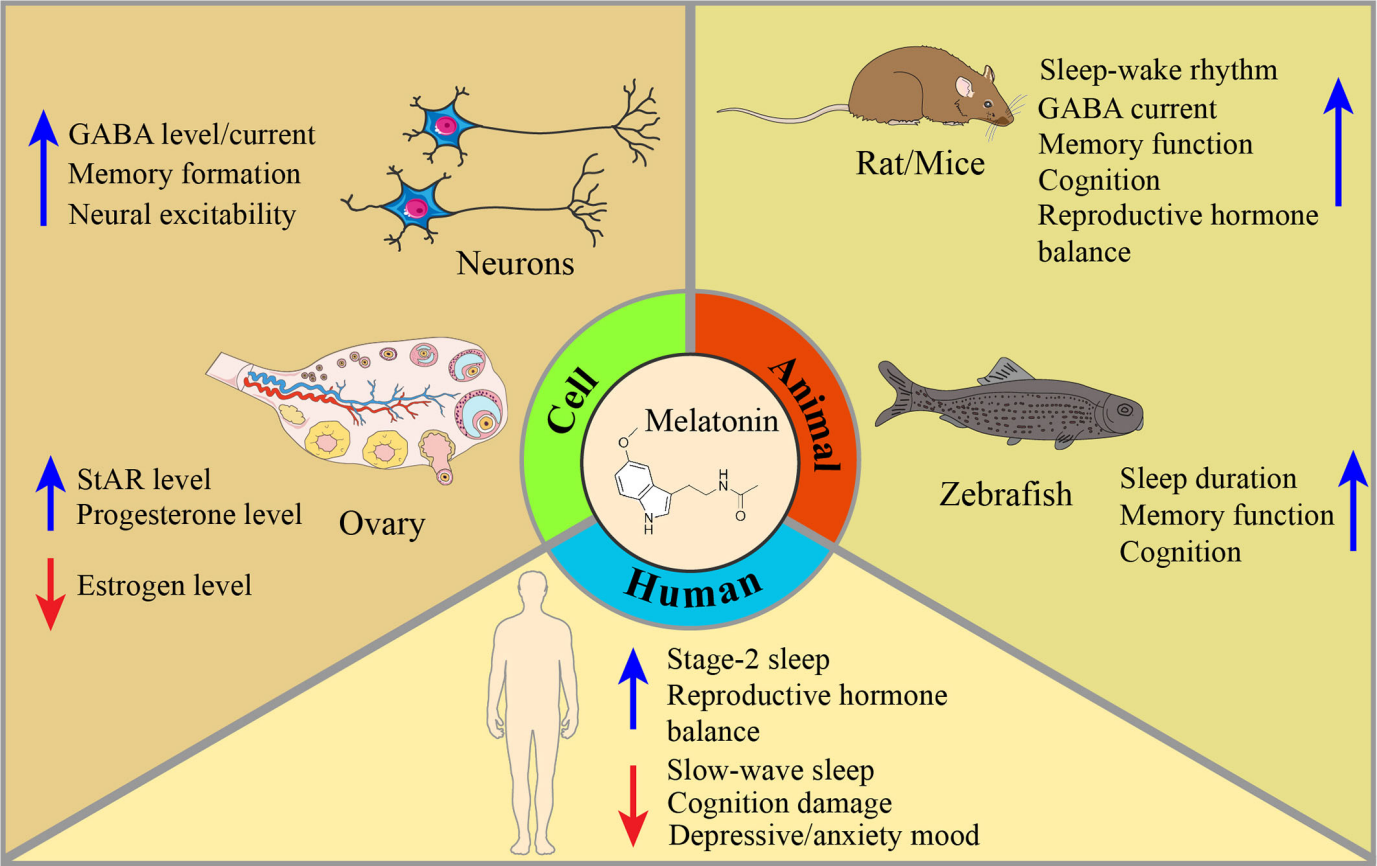
Mechanisms of melatonin restoration in PMS/PMDD-related symptoms, given cells, animals, and humans. Melatonin is involved in many aspects of PMS/PMDD. In cells, melatonin can upregulate GABA levels and its current among neurons, promote memory formation, and increase neuron excitability. To adjust the imbalance of the ovary’s hormones, melatonin can increase the levels of StAR and progesterone, and decrease the level of estrogen. In animals, dysfunction of sleep-wake rhythm, GABA current, memory function, cognition, and reproduction hormone balance have been modulated by melatonin intake. Meanwhile, melatonin’s improvement of sleep, memory, and cognition is also discovered in the zebrafish model. In humans, the effect of melatonin has long been applied in improving sleep quality. In PMS/PMDD-related symptoms, melatonin can restore stage-2 sleep and reproductive hormone balance and reduce slow-wake sleep, cognitive damage, and depressive/anxiety mood derived from PMS/PMDD.

**Figure 2:**
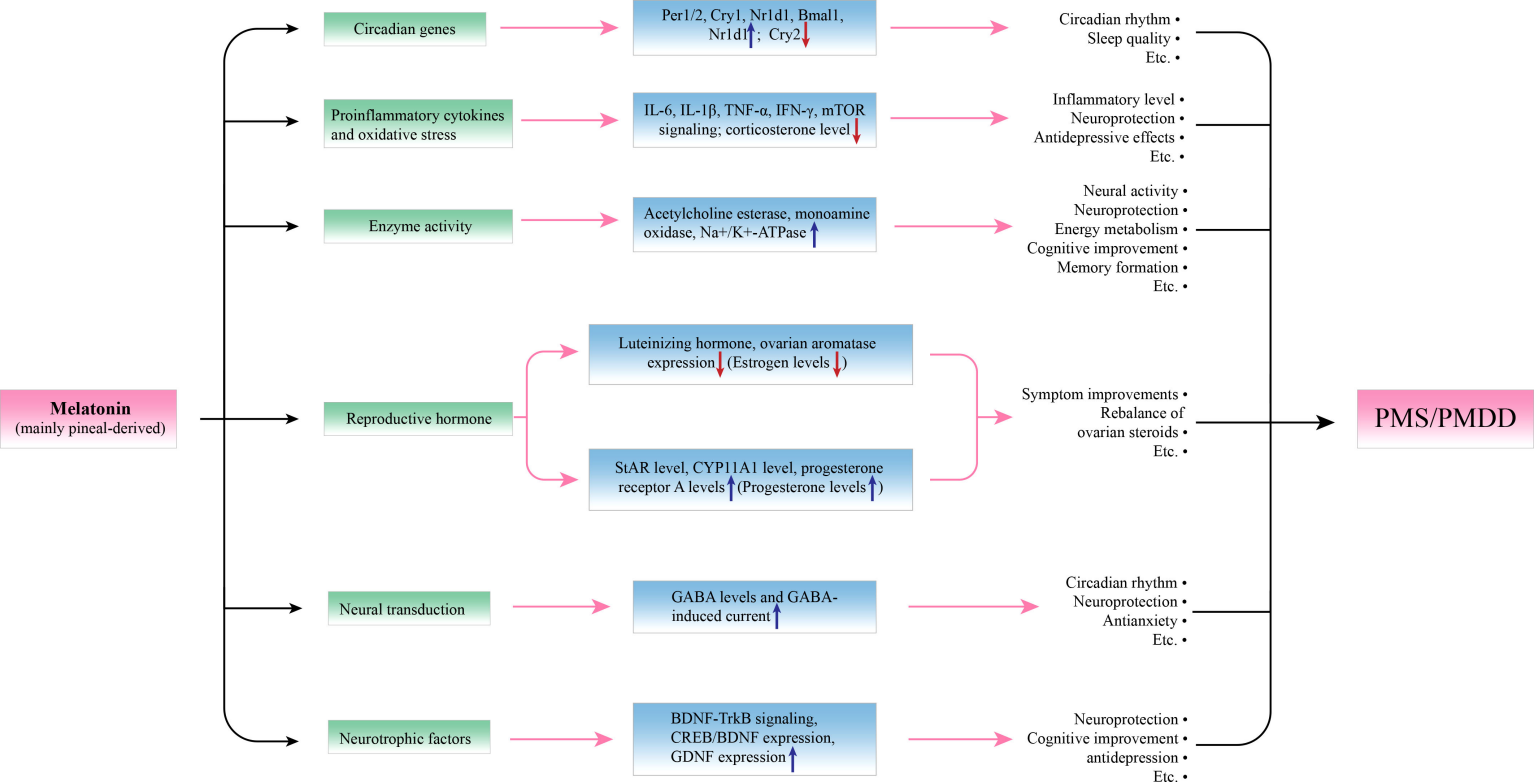
Potential molecular actions of melatonin in modulating premenstrual symptoms. By affecting the expression of relevant molecules, and melatonin can achieve its protective effects in PMS. Per1/2, Cry1/2, and Nr1d1 are circadian genes, melatonin can upregulate their expression to improve circadian rhythm and sleep quality. IL-6, IL-1β, TNF-α, IFN-γ, and mTOR signaling pathways and increased corticosterone levels participate in the inflammation and oxidative stress of PMS, and melatonin can downregulate these processes to play a role in neuroprotection and antidepression. Regarding enzyme activity, melatonin can upregulate the activity of acetylcholine esterase, monoamine oxidase, and Na+/K+-ATPase to promote neural activity, energy metabolism, cognition, and so on. An imbalance of reproductive hormone is seen as a pathogenesis of PMS, melatonin can upregulate the levels of StAR, CYP11A1, and progesterone receptor A and downregulate the levels of luteinizing hormone and ovarian aromatase to reverse the imbalance of the estrogen/progesterone ratio. Moreover, melatonin can promote neural transduction by increasing GABA levels and GABA-induced current, which is associated with better circadian rhythm, neuroprotection, and antianxiety effects. In addition, melatonin can act as a neurotrophic promoter by upregulating the BDNF-TrkB signaling pathway and the expression of CREB/BDNF and GDNF.

In this article, we review the latest literature on PMS and melatonin and their potential relationships from both behavioral and molecular perspectives. Regarding behavioral aspects, accumulating studies have demonstrated that melatonin has an antiPMS function in cell models, animal models and humans (**Figure 1**). Regarding molecular mechanisms, we focused on circadian genes (28), proinflammatory cytokines (IL-6, IL-1β, TNF-α, IFN-γ, and mTOR) (28–30), enzyme activity (31), ovarian hormone estrogen and progesterone (32, 33), gamma-amino butyric acid (GABA) and brain-derived neurotrophic factor (BDNF) alterations in PMS (34, 35) (**Figure 2**, **Tables 2**, **3**). This work attempts to unravel the enigma of how melatonin modulates PMS symptoms through these molecule-related pathways, which may provide a theoretical foundation for melatonin-targeted treatment in PMS and PMDD.

**Table 2:** Selected therapeutic effects of melatonin in PMS/PMDD-associated symptoms.

Disease/model	Species	Associated function	Melatonin effects	Reference
Aanat2-induced melatonin lacking zebrafish	Zebrafish	Sleep	Increased sleep duration	(36)
CRSDs' patients	Human	Sleep	Improved sleep quality	(37)
PMS/PMDD patients	Human	Sleep	Increased stage-2 sleep and reduced slow-wave sleep	(25)
MT1/MT2 knockout mice	Mouse	Sleep	Increased sleep time and improved sleep-wake rhythm	(38)
MT2 knockout mice	Mouse	Sleep	Promoted non-REM sleep and synchronized sleep cycle	(39)
Chronic light-induced model of rats	Rat	Sleep	Improved sleep-wake rhythms (after an MT1/2 receptor agonist: agomelatine)	(40)
PMS/PMDD patients	Human	Mood	Less stress	(41)
Depression model of mice	Mouse	Mood	Inhibited depressive mood	(35, 42)
Patients with depression and anxiety	Human	Mood	Inhibited depression and anxiety (melatonin receptor agonist, ramelteon)	(28)
Isoflurane-induced cognitive deficit model in aged mice	Mouse	Cognition	Improved cognitive function	(29)
Scopolamine-induced cognitive impairment model of mice	Mouse	Cognition	Improved cognitive function	(43)
HT-22 cell line (mouse hippocampus) (in vitro)	Mouse	Memory	Improved memory formation	(44)
Chronic stress model of mice	Mouse	Memory	Improved memory function (after an MT1/2 receptor agonist: agomelatine)	(45)
5-fluorouracil-induced cognitive deficit model in rats	Rat	Spatial memory	Improved spatial memory	(46)
24h-light-induced cognitive deficit model in zebrafish	Zebrafish	Cognition and memeory	Improved memory formation and cognitive function	(47)
Nonylphenol-induced neurotoxicity in rats	Rat	Enzyme activity and cognition	Promoted acetylcholine esterase activity, monoamine oxidase activity, and Na+/K+-ATPase (in frontal cortex and hippocampus); improved cognitive function	(48)
Female mice	Mouse	Hormone	Upregulated ESR-1, ovarian aromatase expression, and progesterone receptor A levels	(49)
Pregnant mice	Mouse	MT receptors and hormone	Upregulated progesterone levels, StAR, and CYP11A1 levels	(31)
Granulosa-lutein cells (human)	Human	MT receptors and hormone	Upregulated StAR levels (MT1and MT2 receptors, PI3K/AKT signaling pathway) and progesterone levels	(33)
Neostriatum of the aware rat	Rat	GABA	Upregulated glutamate and GABA levels (during daytime)	(50)
Hippocampal neurons of rat (in vitro)	Rat	GABA	Increased GABA-induced current (MT receptor independent)	(34)
Hippocampal neurons of rat (in vivo)	Rat	GABA	Promoted GABA(A) receptor function and increased neuron exitability (Nocturnal activation of MT1b receptor)	(51)
GABAA receptor-inhibited rats	Rat	Sleep and GABA	Improved sleep quality and increased GABA receptor function	(52)
Sleep deprevation rats	Rat	Mood and GABA	Decreased Anxiety-like behavior and increased GABAergic/glutamatergic function	(53)
Rats	Rat	GABA	Increased GABA-induced current (in SCN)	(54)
GT1-7 cells in rats	Rat	GABA	Increased GABAA-induced current (with cetrorelix, GnRH antagonist)	(55)
GnRH-EGFP transgenic rats	Rat	GABA	Increased GABAA-induced current (male mainly via MT1; female mainly via MT2)	(56)

**Table 3:** Expression changes and melatonin effects in PMS/PMDD-related symptoms and animal models.

Disease/model	Species	Gene expression/ manifestation	Melatonin effects	References
Patients with a history of depression	Human	Clock, Per1, Bmal1 mRNA↑	Undefined	(57)
Nr1d1 knochdown female model	Mouse	Per1 and Per2↑; Anxiety↓, sociability↑	Undefined	(58)
Idiopathic REM sleep behavior disorder	Human	Clock expression, melatonin secretion↓	Undefined	(59)
Oxygen glucose depreivation/ focal cerebral ischemia	Mouse	PI3K/AKT signaling pathways, cellular survival↓	Bmal1↑ (PI3K/AKT signaling pathways), cellular survival↑	(60)
Insomnia (with depression and anxiety)	Human	Per1 and Per2↓, Cry1↓, Cry2↑, Nr1d1↓	Per1 and Per2↑, Cry1↑, Cry2↓, Nr1d1↑, BDNF↑, pro-inflammatory cytokine levels↓	(28)
Dextran sulphate sodium-induced depression	Rat	TNF-α, IL-1β, and neuroinflammation levels↑	SCFA production (Lactobacillus and Clostridium)↑; neuroinflammation↓	(30)
Isoflurane-induced cognitive deficit model in aged model	Mouse	Melatonin levels↓, mTOR expression↑, proinflammatory cytokines↑ (TNF-α, IL-1β, and IL-6)	mTOR expression↓, proinflammatory cytokines↓ (TNF-α, IL-1β, and IL-6)	(29)
Nonylphenol-induced neurotoxicity	Rat	Acetylcholine esterase activity↓, monoamine oxidase activity↓, Na+/K+-ATPase ↓ (in frontal cortex and hippocampus)	Acetylcholine esterase activity↑, monoamine oxidase activity↑, Na+/K+-ATPase ↑ (in frontal cortex and hippocampus)	(48)
Pinealectomized model	Rat	CYP17A1 expression↑, estrogen levels↑	Undefined	(61)
Pregnant model	Mouse	Undefined	Progesterone levels↑, StAR and CYP11A1 levels↑ (in uterine endometrium)	(31)
Granulosa-lutein cells	Human	Undefined	StAR levels↑ (MT1and MT2 receptors, PI3K/AKT signaling pathway), progesterone levels↑	(33)
Female mice	Mouse	Undefined	ERα↑, progesterone receptor A levels↑; ovarian aromatase expression↓	(49)
MT1 silencing model	Mouse	Follicular apoptosis↑, Bax expression↑, Bcl-2 expression↓; estradiol levels↑, progesterone levels↓ (with follicle stimulating hormone treatment)	Undefined	(62)
Syrian hamster	Hamster	Undefined	LH levels↓, estrogen levels↓ (targeting kisspeptin neurons)	(63)
Bmal1(-/-) female model	Mouse	Progesterone levels↓, steroidogenesis↓	Undefined	(64)
SF1-Bmal1(-/-) female model	Mosue	Progesterone levels↓, steroidogenesis↓, StAR levels↓	Undefined	(65)
Bmal1-silencing luteinzing granulosa cells	Rat	Per1 and Per2↓, StAR levels↓, CYP19A1 and CYP11A1 levels↓; progesterone level↓	Undefined	(66)
Neostriatum of the aware rat	Rat	Glutamate and GABA levels↓ (during daytime)	Glutamate and GABA levels↑ (during daytime)	(50)
Hippocampal neurons of rat (in vitro)	Rat	Undefined	GABA-induced current↑ (MT receptor independent)	(34)
Sleep deprevation rats	Rat	Corticosterone↑, oxidative stress↑	Corticosterone↓, oxidative stress↓	(53)
Mammals	Mammal	GABAergic transmission↓ (in SCN), melatonin levels↓	Undefined	(67)
Normal rat	Rat	Undefined	GABA-induced current↑ (in SCN)	(54)
GT1-7 cells	Rat	MT1 receptor expression↑ (with cetrorelix, GnRH antagonist)	GABAA current↑ (with cetrorelix, GnRH antagonist)	(55)
GnRH-EGFP transgenic rats	Rat	Undefined	GABAA current↑ (male mainly via MT1) and GABAA current↓ (female mainly via MT2)	(56)
PMDD	Human	BDNF levels↑	Undefined	(12)
PMS	Human	BDNF levels↓	Undefined	(68)
Depression model	Mouse	Undefined	BDNF levels↑	(35)
Depression model	Mouse	Undefined	BDNF-TrkB signaling↑ (in hippocampus combing with SSRI: fluoxetine), depression↓	(42)
Scopolamine-induced cognitive impairment model	Mouse	Undefined	BDNF and TrkB expression↑ (in the dentate gyrus, cerebral cortex and hippocampus)	(69)
HT-22 cell line (in hippocampus) (in vitro)	Mouse	Undefined	CREB and BDNF level↑, memory formation↑	(44)
Normal rat	Rat	Undefined	BDNF expression↑, BDNF-positive neurons↑ (after an MT1/2 receptor agonist: agomelatine) (in hippocapus)	(43)
Chronic light-induced model	Rat	Undefined	MT1 receptors↑, BDNF levels↑(after an MT1/2 receptor agonist: agomelatine)	(40)
PTSD model	Rat	Undefined	BDNF levels↑, Per1 and Per2 levels↓ (after an MT1/2 receptor agonist: agomelatine)	(70)
Chronic stress model	Mosue	Undefined	CREB/BDNF expression↑ (after an MT1/2 receptor agonist: agomelatine)	(45)
Depression and anxiety	Human	Undefined	BDNF levels↑, pro-inflammatory cytokine levels↓(MT receptor agonist: ramelteon)	(28)

The symbol ↓ means decreased while the symbol ↑ means increased.

## Melatonin attenuates PMS-induced sleep disturbance

### Sleep disturbance in PMS

PMS women with sleep problems are commonly characterized by sleep-wake rhythm shifts, subjective sleep disturbance, and sleep electroencephalogram trait variations. In a clinical case report, patients with PMS show a delayed sleep rhythm phase in the luteal phase of the menstrual cycle, whereas the phase is advanced in the follicular phase (71). In addition, PMS-related sleep performance can be unrefreshing and insufficient, which causes daytime sleepiness (72, 73). Although the exact mechanism behind PMS and the sleep-wake cycle is still unknown, elevated progesterone levels during the luteal phase might play a key role since they can induce an increase in body temperature, which will ultimately lead to more fragmented sleep (74, 75).

Sleep disturbances during the luteal phase are often a complaint in women with PMS, including poor sleep, insomnia symptoms, and daytime sleepiness (76, 77) (**Table 1**). In a study of 127 medical students with PMS, 96 of them (75.6%) struggled with decreased sleep quality (78). A disrupted circadian rhythm and decreased melatonin can be found in sleep disorders (59). However, diseases such as infertility, polycystic ovary syndrome and dysfunction of the hypothalamic-pituitary-ovarian axis, and even an irregular menstrual cycle can also result in sleep disturbances and self-reported poor sleep since dysmenorrhea and accompanying mood changes (4). In a recent survey investigating the association between menstruating women and sleep quality, PMS patients shows sleep duration declines, and menstruating problems are associated with a higher incidence of insomnia and daytime sleepiness (79). As a result, sleep dysfunction is not limited to PMS, and the assessment of healthy people during the menstrual phase and criteria for those defined as PMS should be considered carefully.

Despite tremendous reports of poorer subjective sleep qualities in the late-luteal phase, women with PMS suggest few objective sleep quality alterations by polysomnographic and quantitative electroencephalogram (EEG) measures. In most cases, objective parameter changes are only found between the follicular and luteal phases. When compared to the follicular phase, shorter rapid eye movement (REM) latency and REM episodes are found during the luteal phase in both PMS patients and controls (80, 81). Interestingly, PMDD patients with depressed mood have EEG patterns similar to those of healthy women but distinguish from the EEG architecture in major depressive disorder (82, 83). Taken together, we mention 2 points here and call on more studies to get in. On the one hand, it is suggested that the absence of altered actual sleep may be a peculiarity in PMS patients with poorer perceived sleep quality. On the other hand, mood changes may not be the only contributor to PMS-induced sleep disturbance; other factors, such as circadian rhythm and melatonin dysfunction can also play a critical role.

### Melatonin restores the altered circadian clock

From the viewpoint of circadian rhythm, PMS-induced sleep disturbance is a reflection of sleep-wake cycle disorder, which can also impact other biological rhythms. In return, the effects of the circadian system on sleep are well recognized, and internal clock impairment is a leading cause of circadian rhythm sleep disorders (CRSDs) (84). Nevertheless, research on the bidirectional relationship between circadian rhythm and sleep in PMS is not sufficient. Shinohara and his colleagues reported body temperature and sleep rhythm changes in a woman with PMS, indicating circadian rhythm and sleep alterations underlying PMS (71). In a laboratory-based study, sleep durations showed consistency with circadian variation (85). A recent cross-sectional study of university students, 78% of whom report social jet lag (different sleep patterns between weekends and school days), shows that circadian system misalignment due to sleep disturbance is significantly associated with menstrual symptoms (86). A reduction in REM sleep sensitive to menstrual phase changes was also found. These findings, although not direct, imply the attribution of circadian rhythm disorder to PMS-induced sleep disturbance.

In PMS, melatonin levels are decreased at both the follicular and luteal phases (24, 87). In the normal menstrual cycle, no alternation is observed in slow wave sleep (SWS). Melatonin secretion appears to be stable during the two menstrual phases. However, in women with PMDD, increased SWS, prolonged objective sleep onset latency, and reduced stage 2 sleep are functionally associated with reduced melatonin levels, and exogenous melatonin can reverse these changes and improve sleep quality (25, 88). These studies provide direct evidence of a potential interaction between melatonin and PMS-induced sleep problems. Herein, although accumulative studies have shown alterations in stage 2 sleep, SWS and REM sleep, blunted melatonin and circadian temperature rhythm in PMS, the exact mechanism of disrupted circadian clock and melatonin secretion in PMS-related sleep problems has not been elucidated.

### Melatonin regulates sleep by targeting the circadian system

Recently, melatonin has been suggested to play a role in sleep circadian regulation and sleep disorder treatment. In zebrafish, melatonin is required for sleep regulation, and sleep duration is significantly reduced by blocking melatonin synthesis (36). Regarding human beings, clinical meta-analyses have shown the therapeutic effects of melatonin for patients with first-degree and secondary sleep disorders (89, 90). Specifically, in CRSDs, melatonin can accelerate entrainment in jet lag, advance phases of circadian rhythms in delayed sleep phase syndrome and increase daytime sleep in shift-work sleep disorder (37). Some melatonin receptor agonists, such as tasimelteon, have already been approved for the treatment of CRSDs (91, 92). These findings provide solid evidence of melatonin’s role in regulating sleep disorders, especially those related to circadian system interruption (**Table 2**).

Melatonin’s effects on sleep disturbance can be found in PMS and other diseases that need differentiation like primary sleep disorder, polycystic ovary syndrome, and primary insomnia (93, 94). For example, an exogenous supply of melatonin can improve sleep quality among women with PMDD (25). Patients with polycystic ovary syndrome, a female reproductive disease with sleep problems, show significantly increased sleep quality after 12 weeks of melatonin supplementation (95). In insomnia, one of the major complaints in women with PMS, melatonin administration can be adopted as a safe adjuvant therapy (96). The therapeutic effects of melatonin may involve the modulation of the internal clock system, as the clock 3111T/C gene polymorphism is associated with altered melatonin levels in women with insomnia (97).

Melatonin’s effects on sleep are diverse depending on the distinct functions of melatonin receptors including MT1 and MT2 (98, 99) (**Table 2**). In MT1 receptor knockout mice, reduced REM sleep and significantly increased non-REM (NREM) sleep are found whereas MT2 receptor knockout mice present a decrease in NREM sleep (38, 39, 100). In addition, distinct localizations of MT1 (mainly on REM-related regions such as the lateral hypothalamus) and MT2 (mainly on NREM-related areas such as the reticular thalamus) receptors also suggest that melatonin plays receptor-specific roles in sleep regulation (101). Moreover, the MT1 receptor can elevate the amplitude of the internal clock to induce the switch from wake to sleep, and the MT2 receptor may be helpful in synchronizing the sleep cycle to the circadian clock (102). However, the distribution of melatonin receptors has not been elucidated in PMS/PMDD. This evidence provides a molecular basis for melatonin, sleep and circadian clock interactions. Given the circadian rhythm changes in PMS, targeting melatonin and its receptors may relieve PMS-induced sleep problems. Well-conducted and large clinical trials are needed. Relevant mechanisms should also be further explored.

## Melatonin improves mood and cognitive function

### Melatonin improves depressed mood

PMS and PMDD patients are characterized by affective disorders such as anxiety and depression. In particular, psychological discomforts such as depression can be the major complaint in women with PMDD (103). As a result, alleviating emotional symptoms is crucial in PMS. Currently, anti-depressants, selective serotonin reuptake inhibitors (SSRIs), have been regarded as the preferred choice in PMS/PMDD treatment (5). However, the dose-dependency and tolerant peculiarities of traditional antidepressants limit their extensive clinical application (104). In recent years, melatonin has increasingly been suggested to regulate emotional symptoms in PMS/PMDD. In PMDD patients, altered circadian rhythms of melatonin are associated with depressed mood (24, 25, 87) (**Table 2**). In a double-blind study, melatonin administration attenuated premenstrual-like symptoms (41). The authors also found that women treated with melatonin exhibited less depression, anxiety, anger, and fatigue than the placebo control group. Some melatonin receptor agonists, such as agomelatine and ramelteon, have been used as novel anti-depressants and have emerged as promising prospects in depression treatment (105, 106). Together, these studies demonstrate melatonin’s regulation of negative mood, providing a basis for melatonin-mediated emotional changes in PMS.

As the synchronizer of melatonin rhythm, the circadian clock is also involved in emotion regulation. To date, major depressive disorder, bipolar disorder and other affective disorders have been associated with a dysfunctional internal clock system (107–109), which might be attributed to clock gene changes. In a mouse model of depression, Period1(Per1) levels are positively correlated with the severity of depression (110). Per2, brain and muscle ARNT-like 1(Bmal1) and nuclear receptor subfamily 1 group D member 1(Nr1d1) changes also induce depression-like behaviors *via* inflammation-related processes (57, 111, 112). However, whether the circadian gene Nr1d1 is associated with depressive changes, especially in females, is not fully understood (58, 111, 113). In cryptochrome circadian regulator 1/2(Cry1/2) knockout mice, elevated anxiety levels are observed compared to those in wild-type mice (114). Given that early wake therapy can improve depressive moods without melatonin alternation and that the suppression effects of light on melatonin are compatible between PMDD and healthy people (21, 87), the circadian system and its coupling pathway dysfunction may be included in the etiology of PMS mood disorders.

Apart from its direct anti-anxiety/depression effects, melatonin can also regulate core circadian genes involved in emotional disorders (**Table 3**). For instance, melatonin increases Per2, Cry1, and Cry2 expression within the anterior pituitary of rats (115). Phase-delayed Per1, Per2, and Cry1 levels are concomitantly reported after melatonin treatment. Moreover, in a seasonal affective disorder mouse model, melatonin supplementation elevates the rhythm amplitudes of Per1, Per2, and Bmal1 in the SCN (116). In addition, scientists have found that melatonin receptor agonists relieved patients’ depression and anxiety with decreased Per1, Per2, Cry1, Cry2 and Nr1d1 expression (28). Melatonin can upregulate Bmal1 level and promote cellular survival by PI3K/AKT signaling pathways (60). These results suggest that melatonin-induced clock gene alterations may be another potential mechanism in PMS-related affective complaints. However, there is still inconsistency inconsistence with limited recognition of the underlying pathways that govern them.

### Melatonin alleviates cognitive impairment

PMS patients often complain of cognitive-related problems in the luteal phase, such as affective lability and a sense of being controlled or overwhelmed. In major depressive disorder, cognitive deficits are proverbially recognized (117), indicating potential cognitive dysfunction in PMS and PMDD. Keenan etal. (6) employed the Trail Making Test B to assess attention capacity differences between PMS and heathy women, and they found that PMS patients exhibited worse performance in the Trails B task during the luteal phase. A recent study compared working memory discrepancies in PMS participants during the follicular phase, which were detected through the N-back task (7). The results suggest that poorer working memory is correlated with increased PMS severity. In PMDD studies, women display poorer N-back task performance in the luteal phase, which is also correlated with PMDD severity, irritability, and functional impairment (118). Admittedly, current studies on PMS/PMDD cognitive alterations are not consistent (119), and some studies fail to report cognitive impairment, especially in PMS patients, which may be due to the decreased severity compared to PMDD. Further studies on cognitive changes in PMS and PMDD should be conducted.

The potential mechanisms of PMS-induced cognitive damage may be attributed to a negative emotional state and dysregulated ovarian steroid secretion. A substantial body of literature has shown that negative moods such as anxiety and depression can result in cognitive impairment (120). Given the characterized affective dysfunction in PMS/PMDD, anti-depression therapy may attenuate cognitive symptoms. Regarding ovarian hormones, estrogen supplementation enhances memory task performance, while progesterone protects cognition after brain injury (121, 122). In addition, estrogen and progesterone receptors are also distributed in brain cognition-related regions, such as the prefrontal cortex and hippocampus. In summary, current studies have demonstrated the emotion and hormone regulation of cognition, although it is plausible whether mood and steroids contribute to cognitive disorders in PMS/PMDD.

Melatonin plays an important role in cognitive regulation (**Table 2**). In zebrafish, melatonin treatment mitigates cognitive disorder, which results from altered circadian rhythm (47). In a 5-fluorouracil-induced cognitive deficits model, melatonin administration reversed rat spatial memory dysfunction (46). Similar effects of melatonin were discovered in an isoflurane-induced mouse model, and the improved cognitive function after melatonin treatment may be mediated through circadian clock resynchronization (29, 123). In clinical studies, melatonin alleviates cognitive disturbance resulting from breast cancer chemotherapy (124)). Recent advances have regarded melatonin as an index of cognitive impairment in elderly individuals (125). Moreover, melatonin has been shown to effectively improve cognitive deficits in AD mouse models (126). These studies from animals to humans demonstrate a direct regulation of cognition by melatonin. In particular, melatonin has been suggested to play a role in cognition through the regulation of circadian hormones such as estrogen. For instance, nonylphenol, an estrogen mimic, can induce cognitive impairment in Wistar rats, whereas melatonin treatment attenuates its neurotoxicity and adverse cognitive impact (48). Together, potential mechanisms by which melatonin can regulate emotion through circadian clock-dependent or clock-independent pathways have emerged, which may be a possible approach to melatonin regulation of PMS/PMDD-induced cognitive impairments.

## Melatonin adjusts ovarian hormone levels

### Decreased estrogen and elevated progesterone

PMS symptoms cyclically occur in the luteal phase and gradually vanish after menstruation, indicating the role of the menstrual cycle in PMS occurrence. In line with this viewpoint, one of the most effective treatments for PMS is ovulation suppression, and the combination of estrogen and progestogen shows a promising effect on attenuating PMS symptoms (9). ESC/E(Z) complex genes and ovarian hormone regulation genes also manifest different expression levels in PMDD patients and controls (8). With the changes in ovarian steroids and the subsequent resting regional cerebral blood flow in PMDD, ESC/E(Z) genes seem to have a greater correlation with brain function and more attention should be given (127). More importantly, extensive studies have demonstrated the effects of estrogen and progestogen on depression, anxiety and other emotional disorders in women (128, 129). As a result, ovulation-related hormones, especially estrogen and progestogen, should be considered key factors in the pathogenesis of PMS.

During the menstrual cycle, estrogen and progestogen showed distinct secretion patterns. After ovulation, estrogen levels have a slight drop and last for 1-2 days. Subsequently, both estrogen and progesterone are increased and peak in the mid-luteal phase and are significantly reduced to their lowest levels before menstruation (130). However, it seemed that the levels of ovarian hormones in PMS patients were not similar to those in normal people. Decreased estrogen and elevated progesterone levels may be characteristic of PMS, albeit with inconsistent findings. Recently, Yen etal. (131) thoroughly explored estrogen and progesterone levels in women with PMDD. They found that PMDD patients have decreased estrogen levels in the luteal phase. Moreover, in women with low estrogen levels, elevated progesterone levels are reported in PMDD patients rather than healthy controls. Other independent studies have also confirmed the role of higher progesterone and lower estrogen in PMDD (132, 133). In addition, ovarian steroid fluctuations indicate their interactions with PMDD symptoms. In premenstrual women, more severe premenstrual symptoms are correlated with a reduction in ovarian estrogen and progesterone levels (134). The estrogen and progesterone changes from low to peak are also associated with PMDD onset (135). Remarkably, estrogen receptor alpha (ERα) has gradually received wide attention in PMDD emotional symptoms. Scientists have discovered associations between ERα single nucleotide polymorphisms, the risk of PMDD and patient psychological traits (136–138). The ESR α-Xbal polymorphism also suggests its modulation of PMDD patient emotion (139). In conclusion, these studies demonstrate ovarian hormone-related changes in PMS and indicate their contribution to PMS symptoms from various perspectives. Notably, the production of reproductive hormones involves extensive sophisticated mechanisms and is under the control of the neuroendocrine system. As a result, ovarian hormone-targeted treatment does not simply correct the abnormalities in estrogen and progesterone levels, and hypothalamus-pituitary-gonadal cyclicity and circadian rhythms should also be considered.

Apart from estrogen and progesterone, PMS also induces oscillations of other endocrine factors whose secretion is dominated by hypothalamic–pituitary axes and regulated by the internal clock, such as cortisol, luteinizing hormone (LH), and follicle-stimulating hormone (FSH). Interestingly, estrogen and progesterone may also potentially contribute to these hormone circadian changes. Estrogen is known for its negative regulation of LH and FSH. Allopregnanolone, a metabolite of progesterone, is elevated in PMDD patients with decreased cortisol levels, implying the control of progesterone in cortisol diurnal secretion (140). Although there is no consensus on cortisol circadian rhythm dysfunction, the cortisol awakening response is significantly attenuated in PMS women, suggesting hypoactivity in the hypothalamus-pituitary axis (141, 142). Other hormone circadian changes are also observed in PMS patients, including elevated mean FSH, advanced FSH rhythms and reduced amplitude of LH pulses in the luteal phase of menstruation (143, 144). In summary, changes in estrogen and progesterone seem unpredictable among PMS/PMDD patients. Studies on upstream hormones of ovarian steroids may help us to understand the mechanisms of PMS/PMDD.

### Melatonin ameliorates the change in ovarian steroids

Accumulating studies have demonstrated the role of melatonin in ovarian physiology and hormone regulation. In addition to the pineal gland, melatonin is also produced by granulosa cells, the cumulus oophorus, and oocytes, leading to higher concentrations of melatonin in follicular fluid than in serum, which may correlate with estradiol and progesterone levels (145). To date, melatonin has been shown to regulate estrogen and progesterone (**Table 2**). Normally, decreased estrogen and increased progesterone levels are commonly reported after melatonin interference (146, 147), which can exert its actions through the regulation of enzymes participating in steroid synthesis. Martínez-Campa and his colleagues found that melatonin inhibits aromatase cytochrome p450 (CYP19) mRNA expression, preventing aromatase-induced estrogen production (32). Under melatonin deprivation (pinealectomy), the expression of CYP17A1, a crucial enzyme in estrogen synthesis, is significantly increased in mice (61). However, melatonin treatment promotes steroidogenic acute regulatory protein (StAR) and CYP11A1 expression in pregnant mice, which catalyzes the synthesis of progesterone (31). Moreover, a recent *in vitro* study also revealed that StAR levels are upregulated after melatonin treatment, leading to elevated progesterone production (33). On the other hand, melatonin can also serve as an estrogen and progesterone receptor modulator. In rats, melatonin treatment contributes to an increase in progesterone receptor A and a decrease in ERα (49). These effects may be mediated *via* the MT1 receptor, as it is significantly increased in the ovary and can downregulate ERα expression (148). A complementary study was conducted by Talpur etal. (62) They demonstrated that MT1 receptor knockout mice exhibit higher estradiol and lower progesterone levels, indicating the role of the MT1 receptor in melatonin’s modulation of ovarian steroids.

Another potential mechanism involving melatonin effects on ovarian hormone levels is circadian clock regulation within both the hypothalamus and ovary. In the SCN, a timing signal from the central circadian clock is indispensable for the LH surge (149). Given melatonin’s modulation of the central clock, it is reasonable to assume that melatonin may regulate LH levels through the SCN-dependent pathway. Kisspeptin neurons within the hypothalamus also regulate LH secretion by detecting estrogen levels and internal clock signals. Although gonadotropin-releasing hormone (GnRH) neurons control reproductive hormone production, melatonin appears to synchronize ovarian hormone secretion through kisspeptin neurons (63). By targeting the kisspeptin neural system, melatonin induces hypofunction of the hypothalamus-pituitary-ovarian axis, which results in decreased LH secretion and ultimately estrogen level reduction. In the ovary, the circadian clock also suggests its modulation to steroids. Direct evidence is that Bmal1 knockout mice show blunted progesterone levels (64, 65). Moreover, critical genes in steroid synthesis, such as CYP19 and StAR, are also tightly controlled by the circadian clock (31, 66). In light of melatonin’s regulation of clock genes, clock-targeted regulation of melatonin may play an important part in estrogen and progesterone production.

In PMS, estrogen and progesterone levels show incongruent results, although the tendency of estrogen reduction and progesterone elevation is increasingly recognized. Interestingly, PMS/PMDD patients often have decreased melatonin production, which is thought to result in elevated estrogen and decreased progesterone levels. These contrarian changes indicate that the regulation of steroids involves multiple biological processes. One potential explanation is that the effects of melatonin reduction are not dominant compared with those of primary ovarian hormone changes, which can also explain the indeterminate estrogen and progesterone changes in PMS. On the other hand, although estrogen and progesterone do not show direct modulation of the circadian system, the SCN, kisspeptin neurons and ovarian circadian clock genes are clearly regulated by hormone feedback (149), indicating the participation of a disrupted internal clock and other endocrine hormones. Studies on melatonin, the circadian system and ovarian steroid interactions in PMS require further exploration.

## Melatonin modulates brain GABA and BDNF

The core symptoms of PMS, such as sleep disturbance, depression, and cognition disorders, suggest the participation of neurobiological processes in PMS and PMDD. To date, the role of the serotonin system in PMS has been elucidated. Serotonin and its receptors are not only involved in the behavioral regulation of PMS but also mediate the actions of estrogen and progesterone on the brain (150). Serotonin-targeted therapy, such as SSRIs, is recommended as the first-line treatment in PMDD. However, other critical brain neurotransmitters and neuromodulators, such as GABA and BDNF, have not been fully investigated in PMS. Hence, we review recent progress on the relationships between GABA/BDNF and PMS/PMDD. Moreover, the latest studies on melatonin-GABA/BDNF interactions are also recapitulated, anticipating the potential neurological pathways of melatonin-PMS interactions.

### GABA system

#### The GABA system participates in PMS-induced behavioral changes

GABA is the main inhibitory neurotransmitter in the central nervous system, participating in sleep, mood and synaptic plasticity regulation. In PMDD patients with mood disorders, GABA concentrations are dramatically reduced in the brain cingulate cortex, medial prefrontal cortex and left basal ganglia compared to healthy women (11). The GABA type A receptors (GABAARs) also suggest their involvement in the pathogenesis of PMS/PMDD. During the high progesterone phase, δ subunit–containing GABAAR (δGABAAR) expression is significantly increased, resulting in decreased neural excitability and anxiety (151). Diminished cyclic changes in δGABAARs conversely attenuate excitability (152). Further studies have demonstrated that ovarian cycle-linked δGABAAR changes in the hippocampus affect γ oscillations, which may contribute to cognitive and memory deficiencies in PMS/PMDD (10).

Potential mechanisms of GABAAR changes may be the response to neuroactive steroid fluctuations such as estradiol and progesterone. In an animal model of PMDD, rapid withdrawal of progesterone increases anxiety-like behaviors and elevates the expression of GABAARs α4 submit (153). Specifically, allopregnanolone, a modulator of GABAARs, is able to enhance GABA effects and is associated with the negative emotions of PMDD. Antagonizing its effects on GABAARs also yielded promising outcomes in attenuating PMS-related symptoms (154). Moreover, in a PMS rat model, a Japanese herbal medicine, Inochinohaha White, attenuates anxious behavior by upregulating GABAAR-mediated signaling *via* an increase in the β2 subunit (155). By targeting the GABA system, sepranolone has indicated its potential in PMDD treatment with good tolerance and safety to a certain extent (156). Taken together, these studies demonstrate the connection between the GABA system and PMS symptoms. GABA-targeted therapy may serve as a beneficial option in PMS treatment.

#### Melatonin regulates PMS Symptoms *via* the GABA System

Although the relationship between melatonin and GABA in PMS is not fully understood, melatonin and GABA system interactions are well established. Melatonin can directly affect the GABA system (**Tables 2**, **3**). Prado and his colleagues found that melatonin disrupts the circadian changes in GABA with increased GABA levels during the daytime (50). In the hippocampus, melatonin shows distinct effects on the GABA system according to the experimental conditions and melatonin concentrations. *In vitro*, melatonin elevates the amplitude and frequency of GABAergic inhibitory currents and transmission (34). In contrast, melatonin can enhance neuron excitability and depress GABAAR expression *via* the activation of MT1 receptors *in vivo* (51).

In addition, the GABA system may also mediate melatonin’s regulation of sleep, emotion, circadian clock and GnRH neurons. In rats, activation of GABAARs decreases the sleep-promoting effects of melatonin (52). In addition, scientists have found that melatonin prevents anxiety-like behaviors by blocking the α2 subunit of GABAARs (53). Moreover, melatonin and circadian system interactions also involve GABA system regulation. On the one hand, by preventing GABA transmission in the SCN, daytime melatonin secretion is significantly increased, indicating that GABA controls SCN inhibition of melatonin (67). On the other hand, melatonin results in SCN neuron excitability by GABA transmission regulation (54). Similar interactions between GABA and melatonin are also found within GnRH neurons. Ishii etal. (55) found that GnRH neurons can downregulate the expression of MT1 and modify melatonin-induced GABA/GABAAR current decreases. Complementary research was conducted, and melatonin was found to modulate GABAAR-mediated GnRH excitability through melatonin receptors (56). In combination with the current literature on the interaction between melatonin and the GABA system, the hypothesis that melatonin may regulate PMS symptoms *via* the modulation of the GABAergic neurotransmitter system is reasonable, and further studies should be conducted.

### BDNF and its signaling pathways

#### BDNF is involved in the etiology of PMS

BDNF, a neural growth factor in the brain, is involved in mood regulation, synaptic plasticity, neuronal growth and survival and other crucial physiological processes. To date, researchers have focused on BDNF changes in PMS patients, and the results indicate the role of BDNF in PMS symptoms and etiopathogenesis. In PMDD patients, higher serum BDNF levels are found in the luteal phase than in normal controls (12). Similar results were found by Oral etal. (13), who showed significantly elevated BDNF levels from the follicular to luteal phase. These differences between PMDD and healthy people may be attributed to compensatory BDNF increases to alleviate depression symptoms during the luteal phase. However, the results on BDNF changes in PMS are not consistent. A contradictory study found that BDNF is significantly decreased in the luteal phase compared to the control, and decreased BDNF levels are often accompanied by negative emotions such as anxiety and depression (68). In addition, BDNF levels are reduced from the follicular to luteal phase. In a PMS rat model, BDNF expression is significantly decreased with the activation of opioid receptors (157). These inconsistent results may indicate distinct neuromodulator response alternations between PMS and PMDD.

The BDNF Val66Met polymorphism, which can reduce BDNF bioactivity, has also been associated with neuropsychic disorders. In mice injected with human BDNF Val66Met, increased anxiety-like behaviors during the estrous phase are observed (158). In PMDD patients, suppressed fronto-cingulate cortex activity is observed and associated with the BDNF Val66Met allele (159). Similar to the GABA system, BDNF intersects with ovarian hormone and PMS neurological hallmarks. Steroid regulation of emotion is also dependent on BDNF and its single-nucleotide polymorphism (160).

#### Melatonin regulates behaviors *via* BDNF-related pathways

With a gradual understanding of the relationship between melatonin and BDNF, despite a paucity of direct evidence, the melatonin-BDNF pathway has been indicated as the potential mechanism of PMS pathogenesis (**Table 3**). According to the current literature, at least two aspects can be summarized to comprehend this possible mechanism. First, melatonin regulates depression, cognition and memory *via* BDNF-related molecular processes. In a mouse model of depression, melatonin displays antidepression effects with the elevation of BDNF levels in the hippocampus (35). Similar effects of melatonin are also observed when combined with fluoxetine treatment, which involves the normalization of hippocampal BDNF-tropomyosin receptor kinase B (TrkB) signaling (42). Apart from emotional changes, cognitive impairment is also common in PMS patients. To date, researchers have found that cognitive damage is attenuated after melatonin treatment by increasing BDNF and TrkB expression in the dentate gyrus, cerebral cortex and hippocampus (69, 161). Melatonin can also regulate long-term memory processes. Sung and colleagues revealed that melatonin can enhance memory formation by increasing both the cyclic adenosine monophosphate response element-binding protein (CREB) and BDNF levels (44), indicating alterations in the CREB-BDNF signaling pathway. Given the characterized depression, cognition and memory lesions in PMDD patients, melatonin regulates PMS *via* BDNF-related pathways.

In addition, pharmacological studies on melatonin receptor agonists have also provided new insight into melatonin-BDNF interactions in PMS. For example, agomelatine, an agonist of MT1 and MT2 receptors, can increase hippocampal BDNF expression and BDNF-positive neurons in rats (43). Agomelatine also corrects disturbed sleep-wake rhythms and sleep architecture through the elevation of MT1 receptors and BDNF levels (40). As a novel antidepressant, agomelatine reduces anxiety-like behaviors and upregulates BDNF levels in the dentate gyrus (70). Per1 and Per2 expression in the SCN is concomitantly decreased. In a chronic stress model, mice showed restored memory function and elevated CREB/BDNF expression after agomelatine application (45). Another melatonin receptor agonist, ramelteon, improves depression and anxiety symptoms by increasing BDNF levels and targeting clock genes (e.g., Per1 and Per2) (28). Taken together, these studies demonstrate BDNF pathway alterations in melatonin’s action on sleep, emotion, cognition and memory, indicating new perspectives on understanding the potential involvement of melatonin in PMS and PMDD.

## Conclusion and outlook

Undoubtedly, cyclical changes in neuroendocrine factors are tightly associated with PMS and PMDD. Ovarian hormone fluctuations during the luteal phase, such as decreased estrogen and elevated progesterone, can induce brain GABA and BDNF changes, which may ultimately contribute to PMS behavioral complaints such as sleep disturbance and mood and cognitive disorders. In this review, we summarize the potential pathogenesis of PMS and the regulation of melatonin on these processes through melatonin receptors and the circadian clock. Current studies on melatonin and PMS are not sufficient. For example, symptoms such as sleep disturbance, depressive mood, and cognitive impairment are not specific and can be easily misdiagnosed with other diseases. Based on previous studies, ovarian steroids seem not specific among PMS patients, and the effect of melatonin supplementation is not clearly clarified. From the view of molecular modulation, what’s the change and relationship between melatonin receptors and PMS are still unknown. As a consequence, studies on melatonin’s role in the female reproductive system should be augmented.

Further research on the relationship between melatonin and PMS may focus on the perspectives described below. First, adequate animal models should be developed to unveil the underlying mechanisms of PMS. Recently, Bellofiore and his colleagues found that spiny mice exhibit PMS-like symptoms and can serve as a preclinical model of PMS (162). However, melatonin secretion patterns and rhythms between nocturnal animals and human beings are not similar, providing another impediment of melatonin-PMS interaction studies. Second, as functional magnetic resonance imaging reveals deficient positive emotion processing during the female luteal phase (163), functional imaging can be adopted to analyze brain activation patterns and neural circuit alterations in PMS. Third, melatonin’s modulation of the hypothalamus-pituitary-ovary (HPO) axis requires further investigation. Although current studies have shown that melatonin regulates estrogen and progesterone levels, since HPO dysfunction is characterized in PMS and regulated by the SCN, kisspeptin neurons and other endocrine factors, does melatonin participate in these regulatory effects? If it participates, what are the possible mechanisms and pathways? These questions should be taken into consideration in future research. Finally, pharmacological studies on melatonin receptor agonists and structural studies on MT1 and MT2 receptors should be conducted. To date, melatonin receptor agonists such as agomelatine and ramelteon are recommended for depression and insomnia treatments, respectively. However, these agonists are nonselective, making it difficult to target MT1 or MT2 receptors specifically to achieve satisfying therapeutic effects. Understanding structural differences between MT1 and MT2 receptors and their different affinities to some compounds may be the foundation of selective melatonin receptor agonist development and application.

## Author contributions

JS contributed to the study concept and design. WY and JZ collected and sorted the literature. WY and YG drew pictures and tables. WY and JZ wrote the first draft. ZW and CD edited and approved the English version of the article. All authors contributed to the article and approved the submitted version.

## References

[1] Rapkin AJ Mikacich JA. Premenstrual dysphoric disorder and severe premenstrual syndrome in adolescents. Paediatr Drugs (2013) 15(3):191–202. doi: 10.1007/s40272-013-0018-4 23529867

[2] Appleton SM. Premenstrual syndrome: Evidence-based evaluation and treatment. Clin Obstet Gynecol (2018) 61(1):52–61. doi: 10.1097/grf.0000000000000339 29298169

[3] Chumpalova P Iakimova R Stoimenova-Popova M Aptalidis D Pandova M Stoyanova M. Prevalence and clinical picture of premenstrual syndrome in females from Bulgaria. Ann Gen Psychiatry (2020) 19:3. doi: 10.1186/s12991-019-0255-1 31969927 PMC6964059

[4] Kloss JD Perlis ML Zamzow JA Culnan EJ Gracia CR. Sleep, sleep disturbance, and fertility in women. Sleep Med Rev (2015) 22:78–87. doi: 10.1016/j.smrv.2014.10.005 25458772 PMC4402098

[5] Lanza di Scalea T Pearlstein T. Premenstrual dysphoric disorder. Med Clin North Am (2019) 103(4):613–28. doi: 10.1016/j.mcna.2019.02.007 31078196

[6] Keenan PA Stern RA Janowsky DS Pedersen CA. Psychological aspects of premenstrual syndrome. I: Cognition and memory. Psychoneuroendocrinology (1992) 17(2-3):179–87. doi: 10.1016/0306-4530(92)90056-d 1438643

[7] Slyepchenko A Lokuge S Nicholls B Steiner M Hall GB Soares CN. Subtle persistent working memory and selective attention deficits in women with premenstrual syndrome. Psychiatry Res (2017) 249:354–62. doi: 10.1016/j.psychres.2017.01.031 28152471

[8] Dubey N Hoffman JF Schuebel K Yuan Q Martinez PE Nieman LK. The ESC/E(Z) complex, an effector of response to ovarian steroids, manifests an intrinsic difference in cells from women with premenstrual dysphoric disorder. Mol Psychiatry (2017) 22(8):1172–84. doi: 10.1038/mp.2016.229 PMC5495630 28044059

[9] Naheed B Kuiper JH Uthman OA O'Mahony F O'Brien PM. Non-contraceptive oestrogen-containing preparations for controlling symptoms of premenstrual syndrome. Cochrane Database Syst Rev (2017) 3(3):Cd010503. doi: 10.1002/14651858.CD010503.pub2 28257559 PMC6464572

[10] Barth AM Ferando I Mody I. Ovarian cycle-linked plasticity of δ-GABAA receptor subunits in hippocampal interneurons affects γ oscillations *in vivo* . Front Cell Neurosci (2014) 8:222. doi: 10.3389/fncel.2014.00222 25157218 PMC4128222

[11] Liu B Wang G Gao D Gao F Zhao B Qiao M. Alterations of GABA and glutamate-glutamine levels in premenstrual dysphoric disorder: A 3T proton magnetic resonance spectroscopy study. Psychiatry Res (2015) 231(1):64–70. doi: 10.1016/j.pscychresns.2014.10.020 25465316

[12] Oral E Ozcan H Kirkan TS Askin S Gulec M Aydin N. Luteal serum BDNF and HSP70 levels in women with premenstrual dysphoric disorder. Eur Arch Psychiatry Clin Neurosci (2013) 263(8):685–93. doi: 10.1007/s00406-013-0398-z 23455589

[13] Oral E Kirkan TS Yildirim A Kotan Z Cansever Z Ozcan H. Serum brain-derived neurotrophic factor differences between the luteal and follicular phases in premenstrual dysphoric disorder. Gen Hosp Psychiatry (2015) 37(3):266–72. doi: 10.1016/j.genhosppsych.2015.03.001 25799087

[14] Matsumoto T Asakura H Hayashi T. Biopsychosocial aspects of premenstrual syndrome and premenstrual dysphoric disorder. Gynecol Endocrinol (2013) 29(1):67–73. doi: 10.3109/09513590.2012.705383 22809066

[15] Yonkers KA Simoni MK. Premenstrual disorders. Am J Obstet Gynecol (2018) 218(1):68–74. doi: 10.1016/j.ajog.2017.05.045 28571724

[16] de Wit AE de Vries YA de Boer MK Scheper C Fokkema A Janssen CAH. Efficacy of combined oral contraceptives for depressive symptoms and overall symptomatology in premenstrual syndrome: pairwise and network meta-analysis of randomized trials. Am J Obstet Gynecol (2021) 225(6):624–33. doi: 10.1016/j.ajog.2021.06.090 34224688

[17] Comasco E Kopp Kallner H Bixo M Hirschberg AL Nyback S de Grauw H. Ulipristal acetate for treatment of premenstrual dysphoric disorder: A proof-of-Concept randomized controlled trial. Am J Psychiatry (2021) 178(3):256–65. doi: 10.1176/appi.ajp.2020.20030286 33297719

[18] Amaral FGD Cipolla-Neto J. A brief review about melatonin, a pineal hormone. Arch Endocrinol Metab (2018) 62(4):472–9. doi: 10.20945/2359-3997000000066 PMC10118741 30304113

[19] Parry BL Berga SL Kripke DF Klauber MR Laughlin GA Yen SS. Altered waveform of plasma nocturnal melatonin secretion in premenstrual depression. Arch Gen Psychiatry (1990) 47(12):1139–46. doi: 10.1001/archpsyc.1990.01810240059010 2244799

[20] Parry BL Berga SL Mostofi N Klauber MR Resnick A. Plasma melatonin circadian rhythms during the menstrual cycle and after light therapy in premenstrual dysphoric disorder and normal control subjects. J Biol Rhythms (1997) 12(1):47–64. doi: 10.1177/074873049701200107 9104690

[21] Parry BL Udell C Elliott JA Berga SL Klauber MR Mostofi N. Blunted phase-shift responses to morning bright light in premenstrual dysphoric disorder. J Biol Rhythms (1997) 12(5):443–56. doi: 10.1177/074873049701200506 9376643

[22] Tosini G Owino S Guillaume JL Jockers R. Understanding melatonin receptor pharmacology: latest insights from mouse models, and their relevance to human disease. Bioessays (2014) 36(8):778–87. doi: 10.1002/bies.201400017 PMC4151498 24903552

[23] Cecon E Oishi A Jockers R. Melatonin receptors: molecular pharmacology and signalling in the context of system bias. Br J Pharmacol (2018) 175:3263–80. doi: 10.1111/bph.13950 PMC6057902 28707298

[24] Shechter A Lespérance P Ng Ying Kin NM Boivin DB. Pilot investigation of the circadian plasma melatonin rhythm across the menstrual cycle in a small group of women with premenstrual dysphoric disorder. PloS One (2012) 7(12):e51929. doi: 10.1371/journal.pone.0051929 23284821 PMC3526531

[25] Moderie C Boudreau P Shechter A Lespérance P Boivin DB. Effects of exogenous melatonin on sleep and circadian rhythms in women with premenstrual dysphoric disorder. Sleep (2021) 44(12). doi: 10.1093/sleep/zsab171 PMC8664575 34240212

[26] Wongprayoon P Govitrapong P. Melatonin receptor as a drug target for neuroprotection. Curr Mol Pharmacol (2021) 14(2):150–64. doi: 10.2174/1874467213666200421160835 32316905

[27] Yong W Ma H Na M Gao T Zhang Y Hao L. Roles of melatonin in the field of reproductive medicine. BioMed Pharmacother (2021) 144:112001. doi: 10.1016/j.biopha.2021.112001 34624677

[28] Satyanarayanan SK Chien YC Chang JP Huang SY Guu TW Su H. Melatonergic agonist regulates circadian clock genes and peripheral inflammatory and neuroplasticity markers in patients with depression and anxiety. Brain Behav Immun (2020) 85:142–51. doi: 10.1016/j.bbi.2019.03.003 30851380

[29] Yuan H Wu G Zhai X Lu B Meng B Chen J. Melatonin and rapamycin attenuate isoflurane-induced cognitive impairment through inhibition of neuroinflammation by suppressing the mTOR signaling in the hippocampus of aged mice. Front Aging Neurosci (2019) 11:314. doi: 10.3389/fnagi.2019.00314 31803045 PMC6877689

[30] Lv WJ Liu C Yu LZ Zhou JH Li Y Xiong Y. Melatonin alleviates neuroinflammation and metabolic disorder in DSS-induced depression rats. Oxid Med Cell Longev (2020) 2020:1241894. doi: 10.1155/2020/1241894 32802257 PMC7415091

[31] Guan S Xie L Ma T Lv D Jing W Tian X. Effects of melatonin on early pregnancy in mouse: Involving the regulation of StAR, Cyp11a1, and ihh expression. Int J Mol Sci (2017) 18(8). doi: 10.3390/ijms18081637 PMC5578027 28749439

[32] Martínez-Campa C González A Mediavilla MD Alonso-González C Alvarez-García V Sánchez-Barceló EJ. Melatonin inhibits aromatase promoter expression by regulating cyclooxygenases expression and activity in breast cancer cells. Br J Cancer (2009) 101(9):1613–9. doi: 10.1038/sj.bjc.6605336 PMC2778514 19773750

[33] Fang L Li Y Wang S Yu Y Li Y Guo Y. Melatonin induces progesterone production in human granulosa-lutein cells through upregulation of StAR expression. Aging (Albany NY) (2019) 11(20):9013–24. doi: 10.18632/aging.102367 PMC6834401 31619582

[34] Cheng XP Sun H Ye ZY Zhou JN. Melatonin modulates the GABAergic response in cultured rat hippocampal neurons. J Pharmacol Sci (2012) 119(2):177–85. doi: 10.1254/jphs.11183fp 22673185

[35] Taniguti EH Ferreira YS Stupp IJV Fraga-Junior EB Mendonça CB Rossi FL. Neuroprotective effect of melatonin against lipopolysaccharide-induced depressive-like behavior in mice. Physiol Behav (2018) 188:270–5. doi: 10.1016/j.physbeh.2018.02.034 29458118

[36] Gandhi AV Mosser EA Oikonomou G Prober DA. Melatonin is required for the circadian regulation of sleep. Neuron (2015) 85(6):1193–9. doi: 10.1016/j.neuron.2015.02.016 PMC4851458 25754820

[37] Spiegelhalder K Nissen C Riemann D. Clinical sleep-wake disorders II: Focus on insomnia and circadian rhythm sleep disorders. Handb Exp Pharmacol (2019) 253:261–76. doi: 10.1007/164_2017_40 28707143

[38] Comai S Ochoa-Sanchez R Gobbi G. Sleep-wake characterization of double MT_1_/MT_2_ receptor knockout mice and comparison with MT_1_ and MT_2_ receptor knockout mice. Behav Brain Res (2013) 243:231–8. doi: 10.1016/j.bbr.2013.01.008 23333399

[39] Ochoa-Sanchez R Comai S Lacoste B Bambico FR Dominguez-Lopez S Spadoni G. Promotion of non-rapid eye movement sleep and activation of reticular thalamic neurons by a novel MT2 melatonin receptor ligand. J Neurosci (2011) 31(50):18439–52. doi: 10.1523/jneurosci.2676-11.2011 PMC6623882 22171046

[40] Tchekalarova J Kortenska L Ivanova N Atanasova M Marinov P. Agomelatine treatment corrects impaired sleep-wake cycle and sleep architecture and increases MT(1) receptor as well as BDNF expression in the hippocampus during the subjective light phase of rats exposed to chronic constant light. Psychopharmacol (Berl) (2020) 237(2):503–18. doi: 10.1007/s00213-019-05385-y 31720718

[41] Kirby AW Clayton M Rivera P Comperatore CA. Melatonin and the reduction or alleviation of stress. J Pineal Res (1999) 27(2):78–85. doi: 10.1111/j.1600-079x.1999.tb00600.x 10496143

[42] Li K Shen S Ji YT Li XY Zhang LS Wang XD. Melatonin augments the effects of fluoxetine on depression-like behavior and hippocampal BDNF-TrkB signaling. Neurosci Bull (2018) 34(2):303–11. doi: 10.1007/s12264-017-0189-z PMC5856716 29086908

[43] Lu Y Ho CS McIntyre RS Wang W Ho RC. Agomelatine-induced modulation of brain-derived neurotrophic factor (BDNF) in the rat hippocampus. Life Sci (2018) 210:177–84. doi: 10.1016/j.lfs.2018.09.003 30193943

[44] Sung JY Bae JH Lee JH Kim YN Kim DK. The melatonin signaling pathway in a long-term memory *in vitro* study. Molecules (2018) 23(4). doi: 10.3390/molecules23040737 PMC6017053 29570621

[45] Gumuslu E Mutlu O Sunnetci D Ulak G Celikyurt IK Cine N. The antidepressant agomelatine improves memory deterioration and upregulates CREB and BDNF gene expression levels in unpredictable chronic mild stress (UCMS)-exposed mice. Drug Target Insights (2014) 8:11–21. doi: 10.4137/dti.S13870 24634580 PMC3948735

[46] Sirichoat A Suwannakot K Chaisawang P Pannangrong W Aranarochana A Wigmore P. Melatonin attenuates 5-fluorouracil-induced spatial memory and hippocampal neurogenesis impairment in adult rats. Life Sci (2020) 248:117468. doi: 10.1016/j.lfs.2020.117468 32105705

[47] Giacomini A Teixeira KH Marcon L Scolari N Bueno BW Genario R. Melatonin treatment reverses cognitive and endocrine deficits evoked by a 24-h light exposure in adult zebrafish. Neurosci Lett (2020) 733:135073. doi: 10.1016/j.neulet.2020.135073 32446774

[48] Tabassum H Ashafaq M Parvez S Raisuddin S. Role of melatonin in mitigating nonylphenol-induced toxicity in frontal cortex and hippocampus of rat brain. Neurochem Int (2017) 104:11–26. doi: 10.1016/j.neuint.2016.12.010 28012845

[49] Bondi CD Alonso-Gonzalez C Clafshenkel WP Kotlarczyk MP Dodda BR Sanchez-Barcelo E. The effect of estradiol, progesterone, and melatonin on estrous cycling and ovarian aromatase expression in intact female mice. Eur J Obstet Gynecol Reprod Biol (2014) 174:80–5. doi: 10.1016/j.ejogrb.2013.11.027 24373455

[50] Marquez de Prado B Castañeda TR Galindo A del Arco A Segovia G Reiter RJ. Melatonin disrupts circadian rhythms of glutamate and GABA in the neostriatum of the aware rat: A microdialysis study. J Pineal Res (2000) 29(4):209–16. doi: 10.1034/j.1600-0633.2002.290403.x 11068943

[51] Stewart LS Leung LS. Hippocampal melatonin receptors modulate seizure threshold. Epilepsia (2005) 46(4):473–80. doi: 10.1111/j.0013-9580.2005.30204.x 15816940

[52] Wang F Li J Wu C Yang J Xu F Zhao Q. The GABA(A) receptor mediates the hypnotic activity of melatonin in rats. Pharmacol Biochem Behav (2003) 74(3):573–8. doi: 10.1016/s0091-3057(02)01045-6 12543221

[53] Zhang L Guo HL Zhang HQ Xu TQ He B Wang ZH. Melatonin prevents sleep deprivation-associated anxiety-like behavior in rats: role of oxidative stress and balance between GABAergic and glutamatergic transmission. Am J Transl Res (2017) 9(5):2231–42. PMC5446506 28559974

[54] Scott FF Belle MD Delagrange P Piggins HD. Electrophysiological effects of melatonin on mouse Per1 and non-Per1 suprachiasmatic nuclei neurones *in vitro* . J Neuroendocrinol (2010) 22(11):1148–56. doi: 10.1111/j.1365-2826.2010.02063.x 20819119

[55] Ishii H Sato S Yin C Sakuma Y Kato M. Cetrorelix, a gonadotropin-releasing hormone antagonist, induces the expression of melatonin receptor 1a in the gonadotropin-releasing hormone neuronal cell line GT1-7. Neuroendocrinology (2009) 90(3):251–9. doi: 10.1159/000231993 19641296

[56] Sato S Yin C Teramoto A Sakuma Y Kato M. Sexually dimorphic modulation of GABA(A) receptor currents by melatonin in rat gonadotropin-releasing hormone neurons. J Physiol Sci (2008) 58(5):317–22. doi: 10.2170/physiolsci.RP006208 18834560

[57] Gouin JP Connors J Kiecolt-Glaser JK Glaser R Malarkey WB Atkinson C. Altered expression of circadian rhythm genes among individuals with a history of depression. J Affect Disord (2010) 126(1-2):161–6. doi: 10.1016/j.jad.2010.04.002 PMC2930045 20471092

[58] Zhao C Gammie SC. The circadian gene Nr1d1 in the mouse nucleus accumbens modulates sociability and anxiety-related behaviour. Eur J Neurosci (2018) 48(3):1924–43. doi: 10.1111/ejn.14066 PMC6113111 30028550

[59] Weissová K Škrabalová J Skálová K Červená K Bendová Z Miletínová E. Circadian rhythms of melatonin and peripheral clock gene expression in idiopathic REM sleep behavior disorder. Sleep Med (2018) 52:1–6. doi: 10.1016/j.sleep.2018.07.019 30195196

[60] Beker MC Caglayan B Caglayan AB Kelestemur T Yalcin E Caglayan A. Interaction of melatonin and Bmal1 in the regulation of PI3K/AKT pathway components and cellular survival. Sci Rep (2019) 9(1):19082. doi: 10.1038/s41598-019-55663-0 31836786 PMC6910929

[61] Maganhin CC Simões RS Fuchs LF Sasso GR Simões MJ Baracat EC. Melatonin influences on steroidogenic gene expression in the ovary of pinealectomized rats. Fertil Steril (2014) 102(1):291–8. doi: 10.1016/j.fertnstert.2014.04.006 24825418

[62] Talpur HS Worku T Rehman ZU Dad R Bhattarai D Bano I. Knockdown of melatonin receptor 1 and induction of follicle-stimulating hormone on the regulation of mouse granulosa cell function. Reprod Biol (2017) 17(4):380–8. doi: 10.1016/j.repbio.2017.10.005 29097083

[63] Revel FG Ansel L Klosen P Saboureau M Pévet P Mikkelsen JD. Kisspeptin: a key link to seasonal breeding. Rev Endocr Metab Disord (2007) 8(1):57–65. doi: 10.1007/s11154-007-9031-7 17380397

[64] Ratajczak CK Boehle KL Muglia LJ. Impaired steroidogenesis and implantation failure in Bmal1-/- mice. Endocrinology (2009) 150(4):1879–85. doi: 10.1210/en.2008-1021 PMC5393263 19056819

[65] Liu Y Johnson BP Shen AL Wallisser JA Krentz KJ Moran SM. Loss of BMAL1 in ovarian steroidogenic cells results in implantation failure in female mice. Proc Natl Acad Sci U.S.A. (2014) 111(39):14295–300. doi: 10.1073/pnas.1209249111 PMC4191810 25225411

[66] Chen H Zhao L Kumazawa M Yamauchi N Shigeyoshi Y Hashimoto S. Downregulation of core clock gene Bmal1 attenuates expression of progesterone and prostaglandin biosynthesis-related genes in rat luteinizing granulosa cells. Am J Physiol Cell Physiol (2013) 304(12):C1131–1140. doi: 10.1152/ajpcell.00008.2013 23596172

[67] Kalsbeek A Garidou ML Palm IF van der Vliet J Simonneaux V Pévet P. Melatonin sees the light: blocking GABA-ergic transmission in the paraventricular nucleus induces daytime secretion of melatonin. Eur J Neurosci (2000) 12(9):3146–54. doi: 10.1046/j.1460-9568.2000.00202.x 10998098

[68] Cubeddu A Bucci F Giannini A Russo M Daino D Russo N. Brain-derived neurotrophic factor plasma variation during the different phases of the menstrual cycle in women with premenstrual syndrome. Psychoneuroendocrinology (2011) 36(4):523–30. doi: 10.1016/j.psyneuen.2010.08.006 20933336

[69] Chen BH Park JH Lee TK Song M Kim H Lee JC. Melatonin attenuates scopolamine-induced cognitive impairment *via* protecting against demyelination through BDNF-TrkB signaling in the mouse dentate gyrus. Chem Biol Interact (2018) 285:8–13. doi: 10.1016/j.cbi.2018.02.023 29476728

[70] Cohen H Zohar J Carmi L. Effects of agomelatine on behaviour, circadian expression of period 1 and period 2 clock genes and neuroplastic markers in the predator scent stress rat model of PTSD. World J Biol Psychiatry (2020) 21(4):255–73. doi: 10.1080/15622975.2018.1523560 30230406

[71] Shinohara K Uchiyama M Okawa M Saito K Kawaguchi M Funabashi T. Menstrual changes in sleep, rectal temperature and melatonin rhythms in a subject with premenstrual syndrome. Neurosci Lett (2000) 281(2-3):159–62. doi: 10.1016/s0304-3940(00)00826-0 10704767

[72] Nicolau ZFM Bezerra AG Polesel DN Andersen ML Bittencourt L Tufik S. Premenstrual syndrome and sleep disturbances: Results from the sao paulo epidemiologic sleep study. Psychiatry Res (2018) 264:427–31. doi: 10.1016/j.psychres.2018.04.008 29704826

[73] Conzatti M Perez AV Maciel RF De Castro DH Sbaraini M Wender MCO. Sleep quality and excessive daytime sleepiness in women with premenstrual syndrome. Gynecol Endocrinol (2021) 37(10):945–9. doi: 10.1080/09513590.2021.1968820 34409910

[74] Sharkey KM Crawford SL Kim S Joffe H. Objective sleep interruption and reproductive hormone dynamics in the menstrual cycle. Sleep Med (2014) 15(6):688–93. doi: 10.1016/j.sleep.2014.02.003 PMC4098663 24841109

[75] Li DX Romans S De Souza MJ Murray B Einstein G. Actigraphic and self-reported sleep quality in women: associations with ovarian hormones and mood. Sleep Med (2015) 16(10):1217–24. doi: 10.1016/j.sleep.2015.06.009 26429749

[76] Gupta R Lahan V Bansal S. Subjective sleep problems in young women suffering from premenstrual dysphoric disorder. N Am J Med Sci (2012) 4(11):593–5. doi: 10.4103/1947-2714.103326 PMC3503382 23181235

[77] Baker FC Lee KA. Menstrual cycle effects on sleep. Sleep Med Clin (2018) 13(3):283–94. doi: 10.1016/j.jsmc.2018.04.002 30098748

[78] Ozisik Karaman HI Tanriverdi G Degirmenci Y. Subjective sleep quality in premenstrual syndrome. Gynecol Endocrinol (2012) 28(8):661–4. doi: 10.3109/09513590.2011.650769 22316208

[79] Xing X Xue P Li SX Zhou J Tang X. Sleep disturbance is associated with an increased risk of menstrual problems in female Chinese university students. Sleep Breath. (2020) 24(4):1719–27. doi: 10.1007/s11325-020-02105-1 32445135

[80] Baker FC Kahan TL Trinder J Colrain IM. Sleep quality and the sleep electroencephalogram in women with severe premenstrual syndrome. Sleep (2007) 30(10):1283–91. doi: 10.1093/sleep/30.10.1283 PMC2266284 17969462

[81] Baker FC Sassoon SA Kahan T Palaniappan L Nicholas CL Trinder J. Perceived poor sleep quality in the absence of polysomnographic sleep disturbance in women with severe premenstrual syndrome. J Sleep Res (2012) 21(5):535–45. doi: 10.1111/j.1365-2869.2012.01007.x PMC3376683 22417163

[82] Parry BL Mendelson WB Duncan WC Sack DA Wehr TA. Longitudinal sleep EEG, temperature, and activity measurements across the menstrual cycle in patients with premenstrual depression and in age-matched controls. Psychiatry Res (1989) 30(3):285–303. doi: 10.1016/0165-1781(89)90020-6 2616693

[83] Parry BL Mostofi N LeVeau B Nahum HC Golshan S Laughlin GA. Sleep EEG studies during early and late partial sleep deprivation in premenstrual dysphoric disorder and normal control subjects. Psychiatry Res (1999) 85(2):127–43. doi: 10.1016/s0165-1781(98)00128-0 10220004

[84] Pavlova M. Circadian rhythm sleep-wake disorders. Continuum (Minneap Minn) (2017) 23(4, Sleep Neurology):1051–63. doi: 10.1212/con.0000000000000499 28777176

[85] Shechter A Varin F Boivin DB. Circadian variation of sleep during the follicular and luteal phases of the menstrual cycle. Sleep (2010) 33(5):647–56. doi: 10.1093/sleep/33.5.647 PMC2864880 20469807

[86] Komada Y Ikeda Y Sato M Kami A Masuda C Shibata S. Social jetlag and menstrual symptoms among female university students. Chronobiol Int (2019) 36(2):258–64. doi: 10.1080/07420528.2018.1533561 30395733

[87] Parry BL Meliska CJ Martínez LF López AM Sorenson DL Hauger RL. Late, but not early, wake therapy reduces morning plasma melatonin: relationship to mood in premenstrual dysphoric disorder. Psychiatry Res (2008) 161(1):76–86. doi: 10.1016/j.psychres.2007.11.017 18789826 PMC3038844

[88] Shechter A Lespérance P Ng Ying Kin NM Boivin DB. Nocturnal polysomnographic sleep across the menstrual cycle in premenstrual dysphoric disorder. Sleep Med (2012) 13(8):1071–8. doi: 10.1016/j.sleep.2012.05.012 22749440

[89] Auld F Maschauer EL Morrison I Skene DJ Riha RL. Evidence for the efficacy of melatonin in the treatment of primary adult sleep disorders. Sleep Med Rev (2017) 34:10–22. doi: 10.1016/j.smrv.2016.06.005 28648359

[90] Li T Jiang S Han M Yang Z Lv J Deng C. Exogenous melatonin as a treatment for secondary sleep disorders: A systematic review and meta-analysis. Front Neuroendocrinol (2019) 52:22–8. doi: 10.1016/j.yfrne.2018.06.004 29908879

[91] Williams WP 3rd McLin DE 3rd Dressman MA Neubauer DN. Comparative review of approved melatonin agonists for the treatment of circadian rhythm sleep-wake disorders. Pharmacotherapy (2016) 36(9):1028–41. doi: 10.1002/phar.1822 PMC5108473 27500861

[92] Nishimon S Nishino N Nishino S. Advances in the pharmacological management of non-24-h sleep-wake disorder. Expert Opin Pharmacother (2021) 22(8):1039–49. doi: 10.1080/14656566.2021.1876665 33618599

[93] Xu H Zhang C Qian Y Zou J Li X Liu Y. Efficacy of melatonin for sleep disturbance in middle-aged primary insomnia: a double-blind, randomised clinical trial. Sleep Med (2020) 76:113–9. doi: 10.1016/j.sleep.2020.10.018 33157425

[94] Fatemeh G Sajjad M Niloufar R Neda S Leila S Khadijeh M. Effect of melatonin supplementation on sleep quality: a systematic review and meta-analysis of randomized controlled trials. J Neurol (2022) 269(1):205–16. doi: 10.1007/s00415-020-10381-w 33417003

[95] Shabani A Foroozanfard F Kavossian E Aghadavod E Ostadmohammadi V Reiter RJ. Effects of melatonin administration on mental health parameters, metabolic and genetic profiles in women with polycystic ovary syndrome: A randomized, double-blind, placebo-controlled trial. J Affect Disord (2019) 250:51–6. doi: 10.1016/j.jad.2019.02.066 30831541

[96] Baglioni C Bostanova Z Bacaro V Benz F Hertenstein E Spiegelhalder K. A systematic review and network meta-analysis of randomized controlled trials evaluating the evidence base of melatonin, light exposure, exercise, and complementary and alternative medicine for patients with insomnia disorder. J Clin Med (2020) 9(6). doi: 10.3390/jcm9061949 PMC7356922 32580450

[97] Semenova NV Madaeva IM Bairova TA Zhambalova RM Sholokhov LF Kolesnikova LI. Association of the melatonin circadian rhythms with clock 3111T/C gene polymorphism in Caucasian and Asian menopausal women with insomnia. Chronobiol Int (2018) 35(8):1066–76. doi: 10.1080/07420528.2018.1456447 29621412

[98] Xie Z Chen F Li WA Geng X Li C Meng X. A review of sleep disorders and melatonin. Neurol Res (2017) 39(6):559–65. doi: 10.1080/01616412.2017.1315864 28460563

[99] Gobbi G Comai S. Differential function of melatonin MT(1) and MT(2) receptors in REM and NREM sleep. Front Endocrinol (Lausanne) (2019) 10:87. doi: 10.3389/fendo.2019.00087 30881340 PMC6407453

[100] Gobbi G Comai S. Sleep well. untangling the role of melatonin MT1 and MT2 receptors in sleep. J Pineal Res (2019) 66(3):e12544. doi: 10.1111/jpi.12544 30586215

[101] Comai S Lopez-Canul M De Gregorio D Posner A Ettaoussi M Guarnieri FC. Melatonin MT(1) receptor as a novel target in neuropsychopharmacology: MT(1) ligands, pathophysiological and therapeutic implications, and perspectives. Pharmacol Res (2019) 144:343–56. doi: 10.1016/j.phrs.2019.04.015 31029764

[102] Touitou Y Bogdan A. Promoting adjustment of the sleep-wake cycle by chronobiotics. Physiol Behav (2007) 90(2-3):294–300. doi: 10.1016/j.physbeh.2006.09.001 17056076

[103] American Psychiatric Association. Diagnostic and statistical manual of mental disorders (DSM-5) . Available at: https://www.psychiatry.org/psychiatrists/practice/dsm (Accessed October 1, 2022).

[104] Marjoribanks J Brown J O'Brien PM Wyatt K. Selective serotonin reuptake inhibitors for premenstrual syndrome. Cochrane Database Syst Rev (2013) 2013(6):Cd001396. doi: 10.1002/14651858.CD001396.pub3 23744611 PMC7073417

[105] Kishi T Nomura I Sakuma K Kitajima T Mishima K Iwata N. Melatonin receptor agonists-ramelteon and melatonin-for bipolar disorder: a systematic review and meta-analysis of double-blind, randomized, placebo-controlled trials. Neuropsychiatr Dis Treat (2019) 15:1479–86. doi: 10.2147/ndt.S198899 PMC6553999 31239683

[106] Gabriel FC de Melo DO Fráguas R Leite-Santos NC Mantovani da Silva RA Ribeiro E. Pharmacological treatment of depression: A systematic review comparing clinical practice guideline recommendations. PloS One (2020) 15(4):e0231700. doi: 10.1371/journal.pone.0231700 32315333 PMC7173786

[107] Johansson C Willeit M Smedh C Ekholm J Paunio T Kieseppä T. Circadian clock-related polymorphisms in seasonal affective disorder and their relevance to diurnal preference. Neuropsychopharmacology (2003) 28(4):734–9. doi: 10.1038/sj.npp.1300121 12655319

[108] Liberman AR Halitjaha L Ay A Ingram KK. Modeling strengthens molecular link between circadian polymorphisms and major mood disorders. J Biol Rhythms (2018) 33(3):318–36. doi: 10.1177/0748730418764540 29614896

[109] Ferrer A Costas J Gratacos M Martínez-Amorós È. Labad J Soriano-Mas C. Clock gene polygenic risk score and seasonality in major depressive disorder and bipolar disorder. Genes Brain Behav (2020):e12683. doi: 10.1111/gbb.12683 32573093

[110] Wang XL Wang DQ Jiao FC Ding KM Ji YB Lu L. Diurnal rhythm disruptions induced by chronic unpredictable stress relate to depression-like behaviors in rats. Pharmacol Biochem Behav (2021) 204:173156. doi: 10.1016/j.pbb.2021.173156 33675839

[111] Nováková M Praško J Látalová K Sládek M Sumová A. The circadian system of patients with bipolar disorder differs in episodes of mania and depression. Bipolar Disord (2015) 17(3):303–14. doi: 10.1111/bdi.12270 25359533

[112] Chen X Hu Q Zhang K Teng H Li M Li D. The clock-controlled chemokine contributes to neuroinflammation-induced depression. FASEB J (2020) 34(6):8357–66. doi: 10.1096/fj.201900581RRR 32329129

[113] Kishi T Kitajima T Ikeda M Yamanouchi Y Kinoshita Y Kawashima K. Association analysis of nuclear receptor rev-erb alpha gene (NR1D1) with mood disorders in the Japanese population. Neurosci Res (2008) 62(4):211–5. doi: 10.1016/j.neures.2008.08.008 18804497

[114] Hühne A Volkmann P Stephan M Rossner M Landgraf D. An in-depth neurobehavioral characterization shows anxiety-like traits, impaired habituation behavior, and restlessness in male cryptochrome-deficient mice. Genes Brain Behav (2020):e12661. doi: 10.1111/gbb.12661 32348614

[115] Jiménez-Ortega V Barquilla PC Pagano ES Fernández-Mateos P Esquifino AI Cardinali DP. Melatonin supplementation decreases prolactin synthesis and release in rat adenohypophysis: correlation with anterior pituitary redox state and circadian clock mechanisms. Chronobiol Int (2012) 29(8):1021–35. doi: 10.3109/07420528.2012.705936 22891630

[116] Nagy AD Iwamoto A Kawai M Goda R Matsuo H Otsuka T. Melatonin adjusts the expression pattern of clock genes in the suprachiasmatic nucleus and induces antidepressant-like effect in a mouse model of seasonal affective disorder. Chronobiol Int (2015) 32(4):447–57. doi: 10.3109/07420528.2014.992525 25515595

[117] Knight MJ Baune BT. Cognitive dysfunction in major depressive disorder. Curr Opin Psychiatry (2018) 31(1):26–31. doi: 10.1097/yco.0000000000000378 29076892

[118] Yen JY Chang SJ Long CY Tang TC Chen CC Yen CF. Working memory deficit in premenstrual dysphoric disorder and its associations with difficulty in concentrating and irritability. Compr Psychiatry (2012) 53(5):540–5. doi: 10.1016/j.comppsych.2011.05.016 21821238

[119] Le J Thomas N Gurvich C. Cognition, the menstrual cycle, and premenstrual disorders: A review. Brain Sci (2020) 10(4). doi: 10.3390/brainsci10040198 PMC7226433 32230889

[120] Iosifescu DV. The relation between mood, cognition and psychosocial functioning in psychiatric disorders. Eur Neuropsychopharmacol (2012) 22 Suppl 3:S499–504. doi: 10.1016/j.euroneuro.2012.08.002 22959115

[121] Kundu P Neese SL Bandara S Monaikul S Helferich WG Doerge DR. The effects of the botanical estrogen, isoliquiritigenin on delayed spatial alternation. Neurotoxicol Teratol (2018) 66:55–62. doi: 10.1016/j.ntt.2018.02.001 29408209 PMC5856646

[122] Lengel D Huh JW Barson JR Raghupathi R. Progesterone treatment following traumatic brain injury in the 11-day-old rat attenuates cognitive deficits and neuronal hyperexcitability in adolescence. Exp Neurol (2020) 330:113329. doi: 10.1016/j.expneurol.2020.113329 32335121 PMC8291368

[123] Song J Chu S Cui Y Qian Y Li X Xu F. Circadian rhythm resynchronization improved isoflurane-induced cognitive dysfunction in aged mice. Exp Neurol (2018) 306:45–54. doi: 10.1016/j.expneurol.2018.04.009 29660304

[124] Palmer ACS Zortea M Souza A Santos V Biazús JV Torres ILS. Clinical impact of melatonin on breast cancer patients undergoing chemotherapy; effects on cognition, sleep and depressive symptoms: A randomized, double-blind, placebo-controlled trial. PloS One (2020) 15(4):e0231379. doi: 10.1371/journal.pone.0231379 32302347 PMC7164654

[125] Kurowska A Bodys-Cupak I Staszkiewicz M Szklarczyk J Zalewska-Puchała J Kliś-Kalinowska A. Interleukin-6 and melatonin as predictors of cognitive, emotional and functional ageing of older people. Int J Environ Res Public Health (2020) 17(10). doi: 10.3390/ijerph17103623 PMC7277775 32455758

[126] Chen C Yang C Wang J Huang X Yu H Li S. Melatonin ameliorates cognitive deficits through improving mitophagy in a mouse model of alzheimer's disease. J Pineal Res (2021) 71(4):e12774. doi: 10.1111/jpi.12774 34617321

[127] Wei SM Baller EB Martinez PE Goff AC Li HJ Kohn PD. Subgenual cingulate resting regional cerebral blood flow in premenstrual dysphoric disorder: differential regulation by ovarian steroids and preliminary evidence for an association with expression of ESC/E(Z) complex genes. Transl Psychiatry (2021) 11(1):206. doi: 10.1038/s41398-021-01328-4 33833224 PMC8032707

[128] Schechter D. Estrogen, progesterone, and mood. J Gend Specif Med (1999) 2(1):29–36. 11252868

[129] Albert KM Newhouse PA. Estrogen, stress, and depression: Cognitive and biological interactions. Annu Rev Clin Psychol (2019) 15:399–423. doi: 10.1146/annurev-clinpsy-050718-095557 30786242 PMC9673602

[130] Mihm M Gangooly S Muttukrishna S. The normal menstrual cycle in women. Anim Reprod Sci (2011) 124(3-4):229–36. doi: 10.1016/j.anireprosci.2010.08.030 20869180

[131] Yen JY Lin HC Lin PC Liu TL Long CY Ko CH. Early- and late-Luteal-Phase estrogen and progesterone levels of women with premenstrual dysphoric disorder. Int J Environ Res Public Health (2019) 16(22). doi: 10.3390/ijerph16224352 PMC6888463 31703451

[132] Thys-Jacobs S McMahon D Bilezikian JP. Differences in free estradiol and sex hormone-binding globulin in women with and without premenstrual dysphoric disorder. J Clin Endocrinol Metab (2008) 93(1):96–102. doi: 10.1210/jc.2007-1726 17956950 PMC2190737

[133] Li Y Pehrson AL Budac DP Sánchez C Gulinello M. A rodent model of premenstrual dysphoria: progesterone withdrawal induces depression-like behavior that is differentially sensitive to classes of antidepressants. Behav Brain Res (2012) 234(2):238–47. doi: 10.1016/j.bbr.2012.06.034 22789402

[134] Ozgocer T Ucar C Yildiz S. Cortisol awakening response is blunted and pain perception is increased during menses in cyclic women. Psychoneuroendocrinology (2017) 77:158–64. doi: 10.1016/j.psyneuen.2016.12.011 28064085

[135] Schmidt PJ Martinez PE Nieman LK Koziol DE Thompson KD Schenkel L. Premenstrual dysphoric disorder symptoms following ovarian suppression: Triggered by change in ovarian steroid levels but not continuous stable levels. Am J Psychiatry (2017) 174(10):980–9. doi: 10.1176/appi.ajp.2017.16101113 PMC5624833 28427285

[136] Huo L Straub RE Roca C Schmidt PJ Shi K Vakkalanka R. Risk for premenstrual dysphoric disorder is associated with genetic variation in ESR1, the estrogen receptor alpha gene. Biol Psychiatry (2007) 62(8):925–33. doi: 10.1016/j.biopsych.2006.12.019 PMC2762203 17599809

[137] Miller A Vo H Huo L Roca C Schmidt PJ Rubinow DR. Estrogen receptor alpha (ESR-1) associations with psychological traits in women with PMDD and controls. J Psychiatr Res (2010) 44(12):788–94. doi: 10.1016/j.jpsychires.2010.01.013 PMC2948969 20172536

[138] Pakharenko L. Effect of estrogen receptor gene ESR1 polymorphism on development of premenstrual syndrome. Georgian Med News (2014) 235):37–41. doi: 10.1016/j.pnpbp.2017.11.013 25416214

[139] Yen JY Wang PW Su CH Liu TL Long CY Ko CH. Estrogen levels, emotion regulation, and emotional symptoms of women with premenstrual dysphoric disorder: The moderating effect of estrogen receptor 1α polymorphism. Prog Neuropsychopharmacol Biol Psychiatry (2018) 82:216–23. doi: 10.1016/j.pnpbp.2017.11.013 29146473

[140] Segebladh B Bannbers E Moby L Nyberg S Bixo M Bäckström T. Allopregnanolone serum concentrations and diurnal cortisol secretion in women with premenstrual dysphoric disorder. Arch Womens Ment Health (2013) 16(2):131–7. doi: 10.1007/s00737-013-0327-1 23329007

[141] Kiesner J Granger DA. A lack of consistent evidence for cortisol dysregulation in premenstrual syndrome/premenstrual dysphoric disorder. Psychoneuroendocrinology (2016) 65:149–64. doi: 10.1016/j.psyneuen.2015.12.009 26789492

[142] Hou L Huang Y Zhou R. Premenstrual syndrome is associated with altered cortisol awakening response. Stress (2019) 22(6):640–6. doi: 10.1080/10253890.2019.1608943 31057066

[143] Reame NE Marshall JC Kelch RP. Pulsatile LH secretion in women with premenstrual syndrome (PMS): evidence for normal neuroregulation of the menstrual cycle. Psychoneuroendocrinology (1992) 17(2-3):205–13. doi: 10.1016/0306-4530(92)90059-g 1359599

[144] Parry BL Hauger R LeVeau B Mostofi N Cover H Clopton P. Circadian rhythms of prolactin and thyroid-stimulating hormone during the menstrual cycle and early versus late sleep deprivation in premenstrual dysphoric disorder. Psychiatry Res (1996) 62(2):147–60. doi: 10.1016/0165-1781(96)02905-8 8771612

[145] Reiter RJ Tamura H Tan DX Xu XY. Melatonin and the circadian system: contributions to successful female reproduction. Fertil Steril (2014) 102(2):321–8. doi: 10.1016/j.fertnstert.2014.06.014 24996495

[146] Chuffa LG Seiva FR Fávaro WJ Amorim JP Teixeira GR Mendes LO. Melatonin and ethanol intake exert opposite effects on circulating estradiol and progesterone and differentially regulate sex steroid receptors in the ovaries, oviducts, and uteri of adult rats. Reprod Toxicol (2013) 39:40–9. doi: 10.1016/j.reprotox.2013.04.001 23591044

[147] Minguini IP Luquetti CM Baracat MCP Maganhin CC Nunes CO Simões RS. Melatonin effects on ovarian follicular cells: a systematic review. Rev Assoc Med Bras (1992) (2019) 65(8):1122–7. doi: 10.1590/1806-9282.65.8.1122 31531613

[148] Molis TM Spriggs LL Hill SM. Modulation of estrogen receptor mRNA expression by melatonin in MCF-7 human breast cancer cells. Mol Endocrinol (1994) 8(12):1681–90. doi: 10.1210/mend.8.12.7708056 7708056

[149] Sen A Hoffmann HM. Role of core circadian clock genes in hormone release and target tissue sensitivity in the reproductive axis. Mol Cell Endocrinol (2020) 501:110655. doi: 10.1016/j.mce.2019.110655 31756424 PMC6962569

[150] Rapkin AJ Akopians AL. Pathophysiology of premenstrual syndrome and premenstrual dysphoric disorder. Menopause Int (2012) 18(2):52–9. doi: 10.1258/mi.2012.012014 22611222

[151] Maguire JL Stell BM Rafizadeh M Mody I. Ovarian cycle-linked changes in GABA(A) receptors mediating tonic inhibition alter seizure susceptibility and anxiety. Nat Neurosci (2005) 8(6):797–804. doi: 10.1038/nn1469 15895085

[152] Stell BM Brickley SG Tang CY Farrant M Mody I. Neuroactive steroids reduce neuronal excitability by selectively enhancing tonic inhibition mediated by delta subunit-containing GABAA receptors. Proc Natl Acad Sci U.S.A. (2003) 100(24):14439–44. doi: 10.1073/pnas.2435457100 PMC283610 14623958

[153] Griffiths J Lovick T. Withdrawal from progesterone increases expression of alpha4, beta1, and delta GABA(A) receptor subunits in neurons in the periaqueductal gray matter in female wistar rats. J Comp Neurol (2005) 486(1):89–97. doi: 10.1002/cne.20540 15834956

[154] Bixo M Johansson M Timby E Michalski L Bäckström T. Effects of GABA active steroids in the female brain with a focus on the premenstrual dysphoric disorder. J Neuroendocrinol (2018) 30(2). doi: 10.1111/jne.12553 29072794

[155] Iba H Watanabe T Motomura S Harada K Uesugi H Shibahara T. A Japanese herbal medicine attenuates anxiety-like behavior through GABA(A) receptor and brain-derived neurotrophic factor expression in a rat model of premenstrual syndrome. J Pharmacol Sci (2021) 145(1):140–9. doi: 10.1016/j.jphs.2020.11.003 33357772

[156] Bixo M Ekberg K Poromaa IS Hirschberg AL Jonasson AF Andréen L. Treatment of premenstrual dysphoric disorder with the GABA(A) receptor modulating steroid antagonist sepranolone (UC1010)-a randomized controlled trial. Psychoneuroendocrinology (2017) 80:46–55. doi: 10.1016/j.psyneuen.2017.02.031 28319848

[157] Song C Xue L. Roles of the µ-opioid receptor and its related signaling pathways in the pathogenesis of premenstrual syndrome liver-qi stagnation. Exp Ther Med (2017) 13(6):3130–6. doi: 10.3892/etm.2017.4374 PMC5450756 28587388

[158] Marrocco J Einhorn NR Petty GH Li H Dubey N Hoffman J. Epigenetic intersection of BDNF Val66Met genotype with premenstrual dysphoric disorder transcriptome in a cross-species model of estradiol add-back. Mol Psychiatry (2020) 25(3):572–83. doi: 10.1038/s41380-018-0274-3 PMC7042769 30356121

[159] Comasco E Hahn A Ganger S Gingnell M Bannbers E Oreland L. Emotional fronto-cingulate cortex activation and brain derived neurotrophic factor polymorphism in premenstrual dysphoric disorder. Hum Brain Mapp (2014) 35(9):4450–8. doi: 10.1002/hbm.22486 PMC4107029 24615932

[160] Borrow AP Cameron NM. Estrogenic mediation of serotonergic and neurotrophic systems: implications for female mood disorders. Prog Neuropsychopharmacol Biol Psychiatry (2014) 54:13–25. doi: 10.1016/j.pnpbp.2014.05.009 24865152

[161] Zhang L Zhang HQ Liang XY Zhang HF Zhang T Liu FE. Melatonin ameliorates cognitive impairment induced by sleep deprivation in rats: role of oxidative stress, BDNF and CaMKII. Behav Brain Res (2013) 256:72–81. doi: 10.1016/j.bbr.2013.07.051 23933144

[162] Bellofiore N Cousins F Temple-Smith P Evans J. Altered exploratory behaviour and increased food intake in the spiny mouse before menstruation: a unique pre-clinical model for examining premenstrual syndrome. Hum Reprod (2019) 34(2):308–22. doi: 10.1093/humrep/dey360 30561655

[163] Dan R Canetti L Keadan T Segman R Weinstock M Bonne O. Sex differences during emotion processing are dependent on the menstrual cycle phase. Psychoneuroendocrinology (2019) 100:85–95. doi: 10.1016/j.psyneuen.2018.09.032 30296706

